# Smart molecules in ophthalmology: Hydrogels as responsive systems for ophthalmic applications

**DOI:** 10.1002/smo.20230021

**Published:** 2024-03-15

**Authors:** Merve Kulbay, Kevin Y. Wu, Doanh Truong, Simon D. Tran

**Affiliations:** ^1^ Department of Ophthalmology & Visual Sciences McGill University Montréal Quebec Canada; ^2^ Division of Ophthalmology Department of Surgery University of Sherbrooke Sherbrooke Quebec Canada; ^3^ College of Arts & Science Case Western Reserve University Cleveland Ohio USA; ^4^ Faculty of Dental Medicine and Oral Health Sciences McGill University Montreal Quebec Canada

**Keywords:** ocular diseases, ocular treatments, smart hydrogels

## Abstract

This comprehensive review delves into a unique intersection of hydrogels as smart molecules and their transformative applications in ophthalmology. Beginning with the foundational definition, properties, and classification of hydrogels, the review explores their synthesis and responsive capabilities. Specific applications examined encompass topical drug delivery, contact lenses, intravitreal drug delivery, ocular adhesives, vitreous substitutes, and cell‐based therapy. A methodical analysis, including an overview of relevant ocular structures and a comparative evaluation of hydrogel‐based solutions against traditional treatments, is conducted. Additionally, potential constraints, translation challenges, knowledge gaps, and research areas are identified. Our methodical approach, guided by an extensive literature review from 2017 to 2023, illuminates the unprecedented opportunities offered by hydrogels, along with pinpointing areas for further inquiry to facilitate their transition into clinical practice.

## Graphical abstract

The provided schematic of the human eye highlights the multifaceted applications of hydrogels in ophthalmology: Topical Administration, targeted at the corneal surface; Soft contact lenses, demonstrating biocompatibility and permeability; Adhesives for wound repair, utilized for ocular surface injuries; Vitreous substitutes, an alternative to the natural vitreous humor; Intravitreal drug delivery systems, addressing retinal diseases; cell‐based therapy, focused on regenerative approaches in the retina.

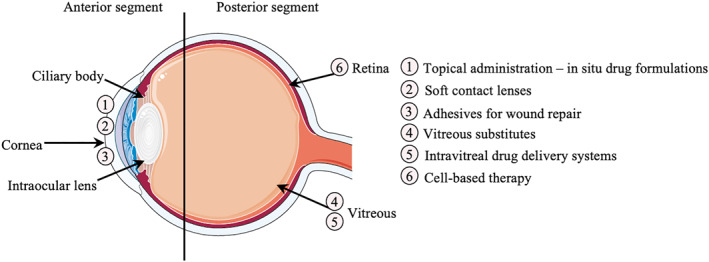

## 1 INTRODUCTION

The eye is a very complex organ with sophisticated anatomy and physiology, where an impairment in its function or integrity can lead to anterior or posterior disorders. According to the World Health Organization, it was estimated that in 2023, at least 2.2 billion people will live with vision impairment and 1.1 billion people will have a preventable or treatable condition. Current treatment options face challenges in terms of drug delivery; ophthalmic drugs have low bioavailability, require frequent administrations, and need to be able to efficiently cross the dynamic ocular barriers. Novel drug delivery technologies have risen over the past decades to leverage these obstacles, including the engineering of smart hydrogels.^[1]^

Smart hydrogels are highly versatile and can be engineered according to the microenvironment they are intended for use. Current research has sought to develop successful applications for smart hydrogels in the setting of topical drug delivery and surgical techniques to overcome these challenges.^[2]^ Given their minimally invasive nature, high bioavailability, and versatile pharmacokinetics, they are sought after as therapeutic tools in the ophthalmology field.^[2]^

In this review, the physicochemical properties of hydrogels will be covered, followed by the novel applications of smart hydrogels for the treatment of ocular pathologies.

## 2 OVERVIEW OF HYDROGELS

**FIGURE 1:**
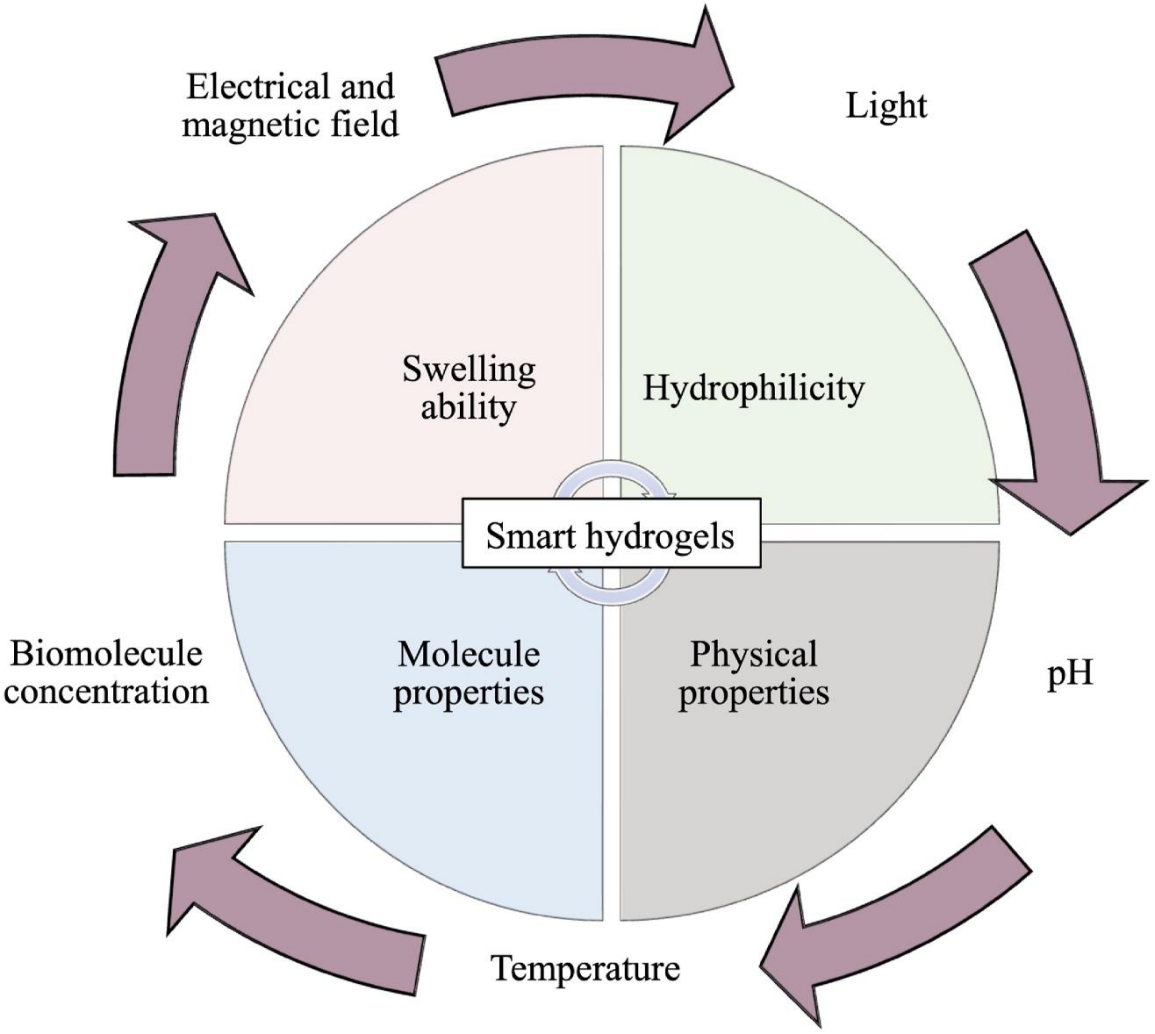
Properties of smart hydrogels. Smart hydrogel properties (*i.e.* swelling ability, hydrophilicity, physical properties, and molecule properties) can be modulated through various external stimuli, such as pH, temperature, biomolecule concentration, light, and electrical and magnetic field. By acting on the smart hydrogel properties through external stimuli, drug delivery into the eye can be achieved in an efficient, biocompatible, and safe manner. Parts of the figure were drawn by using pictures from Servier Medical Art. Servier Medical Art by Servier is licensed under a Creative Commons Attribution 3.0 Unported License (https://creativecommons.org/licenses/by/3.0/).

Hydrogels are composed of three‐dimensional cross‐linked polymers that experience swelling when immersed in an aqueous environment.^[3]^ In the pharmaceutical industry, hydrogels have been studied extensively due to their ability to trap inside their cross‐linked matrix a wide range of substances that vary greatly in size and water‐solubility.^[4]^ Moreover, the “smart” or stimuli‐sensitive feature of the hydrogels constitute another advantage of the gelling system in achieving sustained drug delivery (Figure 1).^[4]^ In particular, exposure to chemical or physical changes in the temperature, pH, or ion concentrations of the body can induce variations in the hydrogel's swelling rate, thereby leading to a change in the drug release profile.^[3]^ The responsiveness of hydrogels is not only limited to physiological changes, but also includes other external stimuli such as the iontophoresis process.^[4]^ Furthermore, the smart hydrogels hydrophilicity, physical properties and molecular properties can also be modulated by external stimuli.

### 2.1 Preparation of hydrogels

Preparation of hydrogels is greatly dependent on the cross‐linking method, which ultimately affects the physicochemical features of hydrogels.^[5]^ Differentiation between physical crosslinking and chemical crosslinking can be determined based on the interaction forces present among the polymeric chains.^[6]^ Physically crosslinked hydrogels, or physical hydrogels, are formed through non‐covalent bonds such as ionic bonds, hydrogen bonds, or chain entanglement.^[7]^ Because of the weak interactions between polymeric chains, physical hydrogels often exhibit reversible responses to external stimuli. In other words, exposure to environmental changes can render the polymeric chains of physical hydrogels vulnerable and disordered.^[7]^ On the other hand, physical hydrogels do not require the use of toxic covalent crosslinking molecules, hence their suitability for use in clinical practices.^[6]^ Although physical hydrogels often dissolve well in the surrounding medium, their longevity is rather limited, with the maximum lifespan being 1 month.^[6]^

Compared to physical hydrogels, chemical hydrogels display superior mechanical stability due to the stronger covalent bonds between polymer chains.^[7]^ To adjust the mechanical properties of chemical hydrogels, small cross‐linking agents such as glutaraldehyde, dopamine, or genipin can be added.^[5]^ Other methods for preparing chemical hydrogels include crosslinking via chemical reactions of functional groups or small molecules, free radical polymerization, or ionizing radiation.^[6]^ In the first scenario, polymeric crosslinking through small molecules or functional groups often involves targeting aldehyde and utilizing Schiff base mechanisms or addition reactions.^[6]^ In free radical polymerization, monomers polymerize upon exposure to a crosslinking agent, hence the development of chemical hydrogels.^[6]^ Meanwhile, crosslinking through ionizing radiation involves the use of light‐sensitive functional groups such as acrylates or azides. In recent years, radiation‐crosslinking has garnered great popularity due to its inexpensive and simple production process.^[6]^ Most importantly, radiation crosslinking does not require extensive use of cross‐linking agents, thereby reducing the toxicity level of chemical hydrogels when applied to living organisms.^[7]^

### 2.2 Classification of smart hydrogels relevant to ophthalmology

#### pH‐sensitive hydrogels

pH‐sensitive gelling systems experience phase changes when there is a change in the environment's pH levels.^[3]^ These polymers have ionic groups which allow them to accept or release protons, hence the mechanism underlying its gelation behavior.^[3]^ In particular, pH‐responsive systems that contain weakly acidic groups on their backbone undergo gelation at pH above their pKa values. As the external pH increases, the polymers become deprotonated, which leads to excess polymer‐polymer repulsion, hence the physical transformation.^[8]^ In contrast, polymers with weak basic moieties become viscous gel as the external pH decreases. This can be attributed to the increase in favorable polymer‐polymer interactions, namely hydrogen bonding among carboxyl groups, that lead to the formation of a gel.^[8]^ Currently, pH‐sensitive polymers that are prevalently used in ocular formulations include carbomer and chitosan.^[3]^

#### Temperature‐sensitive hydrogels

Thermosensitive hydrogels retain a liquid state at ambient temperatures of around 25°C, otherwise known as the hydrogel's lower critical solution temperature (LCST).^[8]^ Transformation into a solid state is observed when the hydrogel is exposed to human physiological temperatures of 34–37°C.^[4]^ Particularly, a rise in the surrounding temperature induces an increase in hydrophobic interactions of the polymers which leads to the formation of micelle networks, hence the observed gelation.^[8]^ Thermosensitive hydrogels can be classified into three groups: negative gel, positive gel, and thermoreversible gel. The negative thermosensitive hydrogel is often used to control the release of proteins in drugs and display contraction when it is heated above critical solution temperature.^[3]^ In contrast, positively thermosensitive hydrogels undergo shrinkage in cooling environments. Thermoreversible hydrogels are often fabricated by embedding copolymers with a water‐soluble co‐monomer and LCST agent.^[4]^ Common examples of thermosensitive systems in ophthalmic formulations include poloxamers, cellulose, xyloglucan, and PLGA‐PEG‐PLGA.^[3]^

#### Ion‐sensitive hydrogels

Ion‐sensitive hydrogels undergo sol‐gel transition in response to a change in ionic concentration of the surrounding environment.^[4]^ In fact, the sol‐gel transition is activated by the attractive forces between oppositely charged species present in the hydrogel and the biological fluid.^[8]^ Moreover, both the gel's viscosity and its sol‐gel conversion rate are determined by the amount or type of cations present in the polymer.^[8]^ For instance, due to electrostatic repulsion, in the presence of monovalent cations (K^+^, Li^+^, Na^+^), gellan gum (GG) hydrogels are weaker and less resistant to flow.^[9]^ Meanwhile, bridges between pairs of carboxylate group and divalent (Ca^2+^, Mg^2+^, Zn^2+^) or trivalent cations (Al^3+^) allow the GG gel to become stiffer and display greater flow resistance.^[9]^ Similarly, in pectin‐based hydrogels, a more heterogeneous structure can be achieved through the steady diffusion of ions from the outside in, leading to limited availability of cations for crosslinking as the calcium front advances.^[10]^ Meanwhile, a more homogeneous gelation can be attained through the steady release of CaCO_3_ and hydrolysis of D‐glucono‐*δ*‐lactone (GDL).^[10]^ In alginate gel, Ca^2+^‐induced gelation occurs through the coordination of Ca^2+^ with six oxygen atoms of the nearby guluronic acid blocks and one to three oxygen atoms of H_2_O, hence producing a highly stable and safe polymeric network.^[11]^ Meanwhile, Fe^3+^‐induced gelation of alginate depends heavily on the binding of Fe^3+^ to the deprotonated carboxyl groups of alginate, thereby producing a gel that is stable yet easily subjected to photoreduction.^[11]^

In ophthalmology, ion‐responsive polymers are ideal for drug delivery because they turn into a viscous gel upon contact with cations that are present in tears.^[5]^ However, this same feature also constitutes several shortcomings of such systems, which includes possible interference by other ionic substances in tear solution and imprecise gelation process.^[3]^ Examples of commonly used ion‐sensitive polymers in clinical settings include sodium alginate, pectin, and GG.^[3]^ It is important to note that GG is an anionic polymer that is responsive to changes in external temperature, pH, and ion concentration.

## 3 OPHTHALMIC APPLICATIONS OF HYDROGELS

**FIGURE 2:**
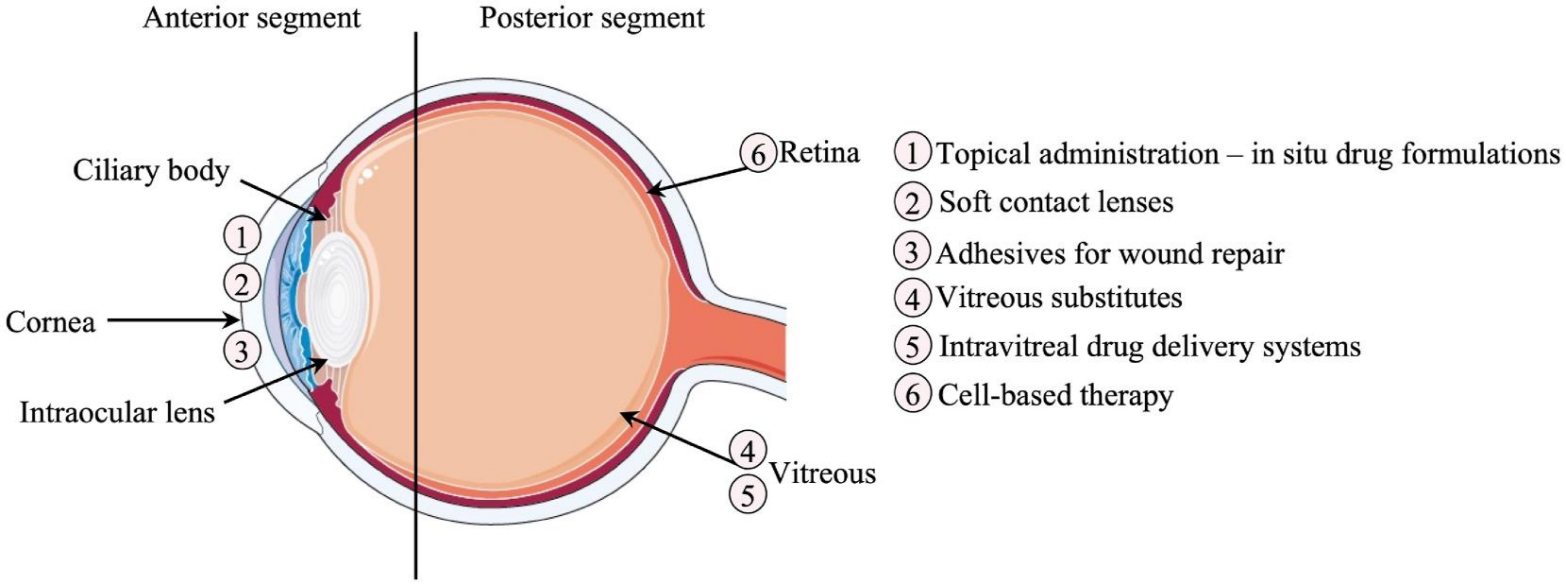
Smart hydrogel applications in ophthalmology. Smart hydrogels can be used for many diverse applications (numbered from 1 to 6) in the treatment of anterior and posterior segment eye diseases. Parts of the figure were drawn using pictures from Servier Medical Art. Servier Medical Art by Servier is licensed under a Creative Commons Attribution 3.0 Unported License (https://creativecommons.org/licenses/by/3.0/).

Smart hydrogels can be applied for the treatment of anterior segment eye diseases (ASED) and posterior segment eye diseases (PSED) given their specific characteristics (Figure 2). A key prerequisite to enable the use of smart hydrogels in ophthalmic applications is transparency. Optical properties of the biocompatible hydrogels depend on their respective refractive index. The main factors that influence hydrogel transparency are polymer concentration, polymer molecular weight, and water content.^[12]^ Therefore, when assessing the possibility of hydrogels in ophthalmic applications, the potential candidates must leverage this obstacle: the ideal thermoresponsive hydrogel must remain transparent under physiological conditions. Although not thoroughly reviewed for each application that will be discussed in the subsequent sections, the optical properties of each smart hydrogel have been characterized and can be found in the given references. In this section, the novel applications of smart hydrogels in the field of ophthalmology will be reviewed.

### 3.1 Ocular surface drug delivery through ophthalmic solutions

**FIGURE 3:**
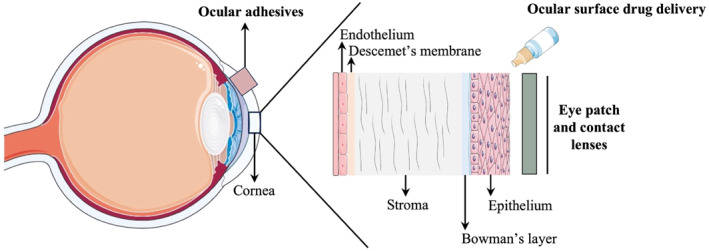
Layers of the cornea: potential applications for smart hydrogels. The cornea is composed of 5 distinct layers: the epithelium, Bowman's layer, stroma, Descemet's membrane, and the endothelium. Smart hydrogels target specific corneal layers through various formulations (*i.e.* ocular adhesives, ocular surface drug delivery, eye patches, and contact lenses). Parts of the figure were drawn using pictures from Servier Medical Art. Servier Medical Art by Servier is licensed under a Creative Commons Attribution 3.0 Unported License (https://creativecommons.org/licenses/by/3.0/).

Due to the eye's complex anatomy, increasing the efficiency of current ocular drug delivery systems remains a difficulty.^[13]^ Particularly, the eyes contain multiple physical barriers that greatly limit the bioavailability of the administered drug (Figure 3).^[13]^ The rapid elimination of drugs through tear formation or eyelid blinking often results in patients having to either utilize eye drops with high drug concentrations or instill drops for multiple intervals throughout the day, leading to low patient compliance.^[14]^ The limitations of conventional eye drops have led many researchers to explore hydrogels as an alternative treatment for many ophthalmic diseases.^[15]^ Specifically, thermosensitive gels have garnered popularity in ophthalmology due to their ability to change from liquid form in ambient conditions to viscous gel at physiological temperature.^[8]^ Ultimately, prolonging the effects of many ophthalmic drugs involves extending the drug retention time in the cornea, which is mostly achieved through the high viscosity nature of the gel.^[14]^ In this section, we will review the most recent advances (*i.e.* within the last 5 years) of smart hydrogels as ocular surface drug delivery carriers (Table 1).

**TABLE 1:** Summary of recent advances in smart hydrogel engineering for topical drug delivery in ophthalmology.^a^

Disease	Active ingredient	Hydrogel composition	Study phase	Formulation	Advantages & considerations	References
Keratitis	Antibiotics	Peptide‐based low‐molecular weight hydrogel	In vitro	Eye drops	Distribute evenly on corneal surfaces; Ease of controlling drug dosage;	[15]
					Can resist tear flushing; Storage conditions render clinical application difficult	
	Ganciclovir	2'deoxyguanosine, ganciclovir, K^+^ ions hydrogel	Ex vivo (human cornea)	Eye drops	Spreads well on ocular surfaces; increased bioavailability; Can resist drainage through tears or blinking	[16]
			In vivo (rabbits)			
	Oxytetracycline	Thermosensitive poloxamer‐N407	In vivo (rabbits)	Eye drops	Mild inflammatory reaction in tested subjects; Easy gelation at physiological temperature; Transparent gels that do not obstruct wearer's vision	[17]
	Bacterial					
	Tannin‐coordinated nanozyme hydrogel + H_2_O_2_	Gelatin hybrid hydrogel	In vitro	Eye drops	Eye drop can penetrate further inside of bacteria and display high antibacterial properties	[18]
	Moxifloxacin	Chitosan/β‐glycerophosphate hydrogel	In vitro	Eye drops	Increased residence time of moxifloxacin in cornea; a more effective treatment against *Staphylococcus aureus* compared to commercially available MOX drops	[19]
			In vivo (rabbits)			
Fungal infection/conjunctivitis/dry eye disease	Bovine lactoferrin and amphotericin‐B	P188‐P407 thermosensitive gel	In vitro	Eye drops	Novel combination of two drugs to treat both ocular inflammations and fungal eye infections;	[17, 18, 20]
			In vivo			
					Could potentially be a safe option for immunocompromised patients	
			Ex vivo (rabbits)			
Corneal scarring	Decorin	Gellan gum	In vitro	Eye drops	Ocular bandage in the form of eye drop that can protect the wound while releasing decorin as anti‐scarring agent	[21]
Dry eye disease (DED)	Resveratrol	Poloxamer 407	In vitro	Eye drops	Increased retention of resveratrol; Antioxidant & anti‐inflammatory effects; Protection against oxidative stress	[22]
	Tacrolimus	Poloxamer 188 & poloxamer 407	In vitro	Eye drops	More effective delivery of drug; 2×/day instillation compared to 3×/day application for conventional Talymus®	[23]
			In vivo (rabbits)			
	Phosphosulindac	Carbopol	In vitro	Eye drops	Longer retention of drug in the cornea; First to report DED treatments with once‐a‐day instillation as compared to 3×/day treatment like previous formulations	[24]
			In vivo (rabbits)			
	Epigallocatechin gallate	Gelatin; PNIPAAm; lectin	In vitro	Eye drops	Effect of a single instillation of eye drop last 14 days	[25]
			In vivo (rabbits)			
	‐	Photothermal gelatin	In vitro	Eye patch	Can adjust secretion of natural tears based on intensity of external light; More environmentally friendly; more convenient for normal eye use;	[26]
			In vivo (rabbits)		Deficient tear film lipid layer remains an issue	
Glaucoma	Nebivolol	P507 & P188 thermoresponsive polymers; kappa‐carrageenan ion‐sensitive polymers	In vitro	Eye drops	Sustained drug release; Minimize systemic side effects	[27]
			In vivo (rabbits)			
	Pilocarpine	Thermoresponsive PNIPPAm; gelatin; GSH	In vitro	Eye drops	Biocompatible; Effects of drug lasted for 14 days after a single topical instillation	[28]
			In vivo (rabbits)			
	Timolol	Chitosan/HTTC	In vitro	Eye drops	Transparent; Easy adjustment of thermosensitivity level; Fast gelation time	[29]
Corneal chemical burn	Indomethacin	Thermoresponsive poloxamer/hyaluronic acid	In vitro	Eye drops	Accelerate corneal wound healing; exact molecular mechanism of expediting wound healing remains unknown	[30]
			In vivo (rabbits)			
Uveitis	Adalimumab	Low‐deacetylated chitosan/β‐glycerophosphate	In vitro	Eye drops	Enhance drug permeation rate in anterior chamber; potential non‐invasive treatment for uveitis	[31]
			In vivo			
Age‐related macular degeneration	(3β, 25R)‐spirost‐5‐en‐3‐ol (Dio)	Calcium alginate	In vivo (rhesus monkeys)	Eye drops	pH‐sensitive release of drug; Novel non‐invasive treatment of AMD; High antiangiogenic activity	[32]
Covid‐19	Remdesivir	Thermosensitive Poly(DHSe/PEG/PPG Urethane)‐Based Hydrogel	In vitro	Eye drops	Novel treatment to disrupt spreading of SARS‐CoV‐2 through ocular passages	[33]
			In vivo (rabbits)			

^a^ Published original research manuscripts from 2017 to 2023.

#### Keratitis

Keratitis (*i.e.* inflammation of the cornea mostly affecting the epithelium) is conventionally treated using antibiotics.^[34]^ However, antibiotic eye drops often result in low patient compliance as the antibiotics must be instilled frequently at a high concentration.^[34]^ Failure to comply with these requirements could result in bacterial resistance and ineffective disease management. To address these limitations, Pan et al. proposed the use of peptide‐based low‐molecular weight hydrogel as a carrier for antibiotics.^[15]^ The new eye drop formulation not only allows the drug to spread evenly on the corneal surface but also resists nasolacrimal drainage; therefore, fewer administrations are needed throughout the day. Additionally, control of ocular irritation as well as retention of antibiotics can be easily achieved through adjusting the hydrogel elasticity so that it matches the ocular surface microenvironment. Despite the promising findings, storage conditions of 4°C greatly hinder the widespread clinical use of this gelling formulation.

**FIGURE 4:**
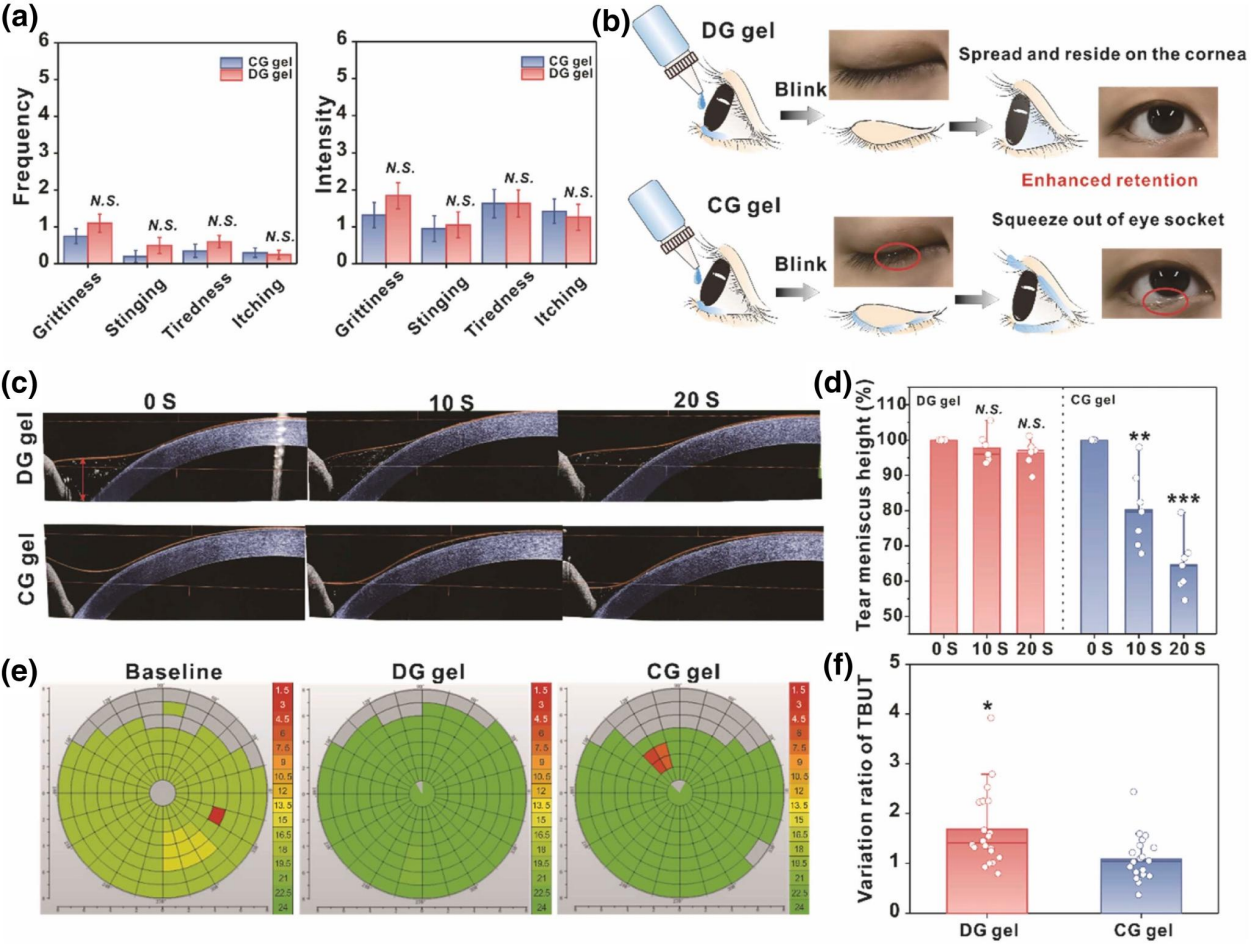
Evaluation of DG gel in its comfortability and corneal delivery. (a) Frequency and intensity scores of grittiness, stinging, tiredness, and itching in 20 humans following topical application of DG and CG gel, respectively. (b) Schema of DG and CG gel on human ocular surface. (c) Optical coherence tomography (OCT) images of the human ocular surface at 0, 10, and 20s post‐instillation of the DG gel and CG gels. (d) Height of tear meniscus obtained at 0, 10, and 20 s post‐instillation of the DG gel and CG gels, *n* = 7. (e) Immediate tear break‐up time test (BUT) images following DG and CG gel application. Red indicates 0∼6 s, yellow indicates 6∼12 s, and green indicates 12∼2 s. (f) Changes in BUT values following DG and CG gel application, *n* = 17. Comparisons between the two gels were performed, not statistically significant with *p* > 0.05. Reprinted with permission.^[16]^ Copyright 2023, with permission from Elsevier.

Commonly proposed hydrogel materials for the treatment of Herpes Simple Keratitis include thermosensitive Pluronic F127 and Carbomer.^[35]^ However, the inability to control the gelation rate upon exposure to the ocular surface renders the long‐term use of such gelling systems impractical. Moreover, current polymer‐based gelling systems often create thick gel layers on corneal surfaces that are easily drained through eyelid blinking.^[16]^ The repulsive forces within the Carbomer gel matrix also represent another disadvantage in that they are greatly susceptible to disintegration upon exposure to ions in tears.^[16]^ Hu et al. overcame the aforementioned drawbacks by constructing a supramolecular gel consisting of small molecules, ganciclovir and 2′‐deoxyguanosine (DG gel) (Figure 4).^[16]^ Essentially, the uniform distribution of the viscoelastic DG gel across the ocular surface allows it to resist drainage following eyelid blinking. Moreover, compared to conventional ganciclovir gels (CG gel), the DG gel demonstrated superior antiviral activity because of the sustained corneal drug retention and was found to be unobstructive to the patient's vision.

Elhabal et al. combined both bovine lactoferrin and amphotericin‐B loaded in triblock polymers PLGA‐PEG‐PEI nanoparticles by immersing the drugs‐loaded nanoparticles in P188‐P407 thermosensitive gel.^[20]^ Ultimately, the innovative combination of drugs allows for higher corneal permeation and sustained release of active ingredients. Abbas et al. suggested the use of thermosensitive poloxamer‐N407 to prolong the effects of oxytetracycline against *P.aeruginosa* infection.^[17]^ Although some mild inflammatory reactions were detected, the application of the new formulation to rabbits' eyes prompted a stronger response of oxytetracycline in regenerating the conjunctival epithelial lining when compared to other commercially available drugs. Moreover, the transparent apperance of the gel prevents it from interfering with the patient's vision. Meanwhile, Wang et al. reported efforts in treating non‐ and drug‐resistant *P. aeruginosa* using nanozyme hydrogel eye drops.^[18]^ Although nanozymes have been widely used for bacterial infections in the skin and stomach, their application in ocular infections remains limited. In this study, in vitro administration of the tannin‐coordinated nanozyme composite‐based hybrid hydrogel eye drop revealed deep penetration inside the bacteria with peroxidase‐like performance that can prevent drug‐resistant bacteria from developing further.

Asfour et al. engineered a thermosensitive chitosan/β‐glycerophosphate hydrogel as an ocular delivery system for moxifloxacin (MOX).^[19]^ The ideal gelation temperature of the hydrogel system was found to be 35°C, which closely matched the temperature of the conjunctival sac. Compared to plain MOX and commercially available MOX eye drops, the hydrogel eye drops demonstrated a greater potency against *Staphylococcus aureus* in rabbits. This could be attributed to the prolonged corneal retention time of the hydrogel eye drop. Notably, even with only 0.25% drug content, the hydrogel eye drop still outperforms the 0.5% MOX commercial eye drop in inhibiting bacterial growth, indicating the potentiality for reduced dosage. However, it remains unknown whether the temperature‐sensitive gel can fully retain its properties when used in tropical regions.

Overall, the use of smart hydrogels as antibiotic carriers in the treatment of infectious keratitis has demonstrated its great potential. Thermoresponsive hydrogels can overcome the nasolacrimal drainage system and exhibit higher bioavailability within the ocular surface. However, a major drawback limits their potential application: drug stability. Current ophthalmic solutions have great shelf stability and can be easily used by patients over their treatment course. However, the ability of smart hydrogels to retain their properties in temperature variable locations has not yet been elucidated.

#### Corneal scarring

**FIGURE 5:**
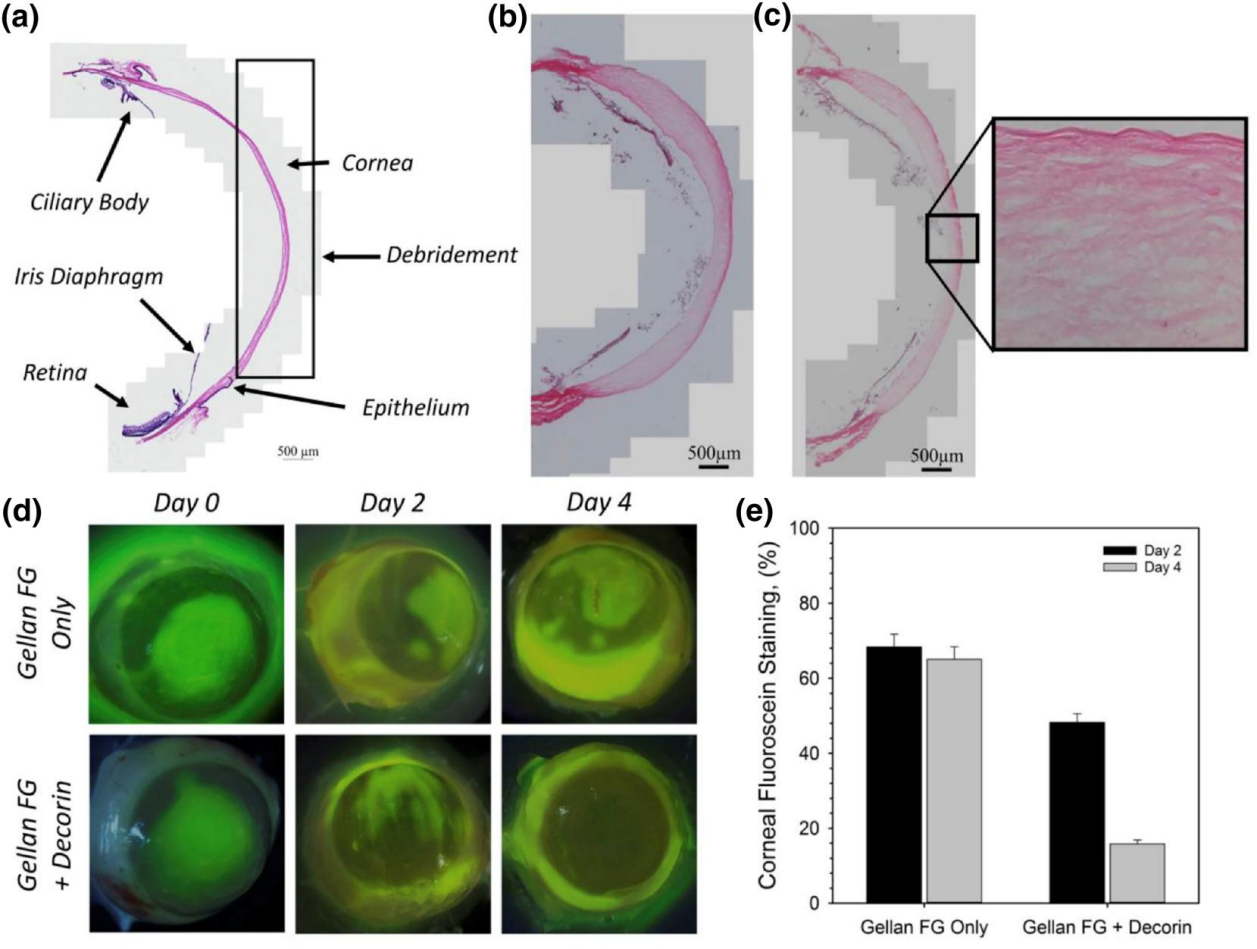
The histological image of stained rat's cornea using an ex vivo corneal wound healing model. Reprinted with permission.^[21]^ Copyright 2023, with permission from Elsevier. License: http://creativecommons.org/licenses/by/4.0/.

Conventional treatment to prevent corneal scarring following infectious diseases or corneal trauma involves applying an amniotic membrane to the ocular surface.^[36]^ While amniotic membrane represents an effective therapeutic choice, such treatment is not accessible to all patients for various reasons such as ethical issues, high treatment cost, disease transmission risk, and strict storage conditions.^[37]^ To address these limitations, Chouhan et al. developed a GG hydrogel eye drop that can deliver Decorin, an anti‐scarring agent (Figure 5).^[21]^ Essentially, the eye drop serves as an ocular bandage that can protect the wound and deliver anti‐scarring molecules. Sustained delivery of decorin for up to 2 h on a rodent's eye is observed through the dynamic transition of the polymer between solid‐liquid‐solid states. Administration of the decorin‐containing eye drop on an organ culture model also showed elevated re‐epithelialization 4 days post‐treatment.

#### Corneal chemical burn

In the treatment of corneal chemical burn, indomethacin, a nonsteroidal anti‐inflammatory (NSAID) drug, provides potent anti‐inflammatory effects through the inhibition of cyclooxygenase‐2 enzymes (COX‐2).^[38]^ However, indomethacin's hydrophobicity and inability to tolerate alkaline environments renders the use of it as a topical drug difficult.^[39]^ Augusto de Castro et al. addressed the issue of bioavailability by creating a poloxamer‐based thermoresponsive gel consisting of both hyaluronic acid (HA) and indomethacin.^[30]^ Essentially, at room temperature indomethacin is trapped inside the nanomicelle. The aggregation of nanomicelles occurs when the surrounding temperature increases. Consequently, sustained release of indomethacin was achieved for 24 h. Although the novel in situ gel was observed to expedite the recovery of corneal wounds, the exact molecular mechanism underlying the acceleration of corneal wound healing remains unclear.

#### Dry eye disease

Dry eye disease (DED) is characterized by hyperosmolarity and tear film instability, which leads to neurosensory abnormalities as well as inflammation of the ocular surface and lacrimal gland.^[40]^ In clinical settings, DED is often classified into one of two divisions: aqueous‐deficient DED caused by limited lacrimal gland output or hyper‐evaporative DED caused by meibomian gland deficiency.^[41]^ The backbone for DED treatment is artificial tears.^[42]^ However, artificial tears are not a perfect replacement of natural tears because they lack many essential components (*e.g.* electrolytes, enzymes, or antioxidants) that are present in natural tears.^[43]^ Moreover, many commercially available artificial tears contain preservatives that could cause serious damage to ocular cells.^[44]^ Meanwhile, other therapeutic options for DED‐like infrared eye masks also possess several limitations such as needing to be recharged, disposable, and inconvenient for daily wear.^[26]^ In general, most thermal therapeutic options for DED, ranging from self‐applied warm compresses to in‐office treatment, are only able to provide temporary relief due to their time‐consuming regimen and low patient compliance.^[45]^

To this end, Pang et al. developed a mini‐eye patch made of photothermal Gelatin and gold nanorods for the treatment of DED.^[26]^ Particularly, upon exposure to external light, the hydrogel eye patch transforms light energy into heat and prompts the lacrimal gland to secrete more tears, thereby alleviating dryness in the eyes. The patch also has a built‐in temperature‐sensitive ink that changes color when the patch temperature gets too high, indicating users of the need for the eyes to rest. Although the eye patch represents a convenient and environmentally friendly alternative for DED therapy, future studies are needed to evaluate whether the lacrimal gland will become permanently dependent on the eye patch for tear production after extended use. Furthermore, dysfunctional tear film lipid layers in DED remain an issue that warrants additional investigation.

De Luca et al. presented a novel use of thermosensitive Poloxamer 407 as a carrier for resveratrol (RSV).^[22]^ Although previous studies have highlighted the potential of RSV as a therapy for DED, its hydrophobicity and limited ocular bioavailability hinder it from being widely used in clinical settings.^[46]^ The present study resolves these challenges by creating RSV‐loaded PLGA‐PEI nanoparticles that are immersed in Poloxamer 407 hydrogel.^[22]^ The new gelling formulation not only prolongs the residence time of RSV in the cornea but also promotes the immune response. Specifically, because RSV is known to have potent antioxidant and anti‐inflammatory properties, the incorporation of such an ingredient in ophthalmic medication could reduce oxidative stress. As DED is often associated with an overexpression of reactive oxygen species (ROS) in tears, this new formulation offers a promising solution for opposing ROS‐induced ocular damage and reducing the frequency of topical instillation throughout the day. Conversely, Wang et al. explored the use of Poloxamer 188 and Poloxamer 407 as a carrier for Tacrolimus.^[23]^ Although there are commercially available DED eye drops containing Tacrolimus, such formulations often result in moderate ocular irritation following administration.^[47]^ Moreover, the bioavailability of Tacrolimus is greatly limited as it is both hydrophobic and has a high molecular weight (804 g/mol).^[48]^ This present study aims to not only construct an ophthalmic formulation that can extend the residence time of Tacrolimus in the cornea but also improve biocompatibility. Precisely, retention time on the cornea is achieved by interacting the positively charged cationic nanoemulsions (CNE) with the negatively charged ocular surface mucin. Meanwhile, low viscosity of the CNE is resolved by adding poloxamers, which can transform from liquid to a viscous gel at critical temperatures. Tests on dry eye rabbit models showed that CNE formulations modified by thermosensitive poloxamers have a precorneal retention time of 50 min, which is longer than that of CNE alone (25 min). DED was completely resolved after 5 days of treatment, with application occurring twice/day as compared to thrice/day for traditional Talymus®. Moreover, in groups treated with the new CNE formulation, no incidence of ocular irritation was noted. Despite the optimistic outcomes, further in vivo studies must be conducted on humans to ensure that the gelling formulation is highly compatible with human's ocular anatomy.

Huang et al. developed an ophthalmic formulation consisting of Carbopol hydrogel and Phospho‐sulindac (PS), a newly discovered molecule with anticancer and anti‐inflammatory features.^[24]^ In an unpublished data, anti‐inflammatory potency was achieved only with a high concentration of 2.2% PS and thrice/day topical application.^[24]^ The systemic safety of the drug along with the inconvenience of thrice/day instillation constitutes two major concerns of past formulations for DED treatment. In this study, tests of the novel hydrogel formulation were performed on both New Zealand white (albinos) and Dutch‐belted black rabbits (pigmented) to mimic the binding affinity of some drugs for melatonin in human ocular tissues. HPLC results indicated that the hydrogel formulation has limited systemic impact with over 95% of PS found in the anterior eye, decreasing the likelihood of unwanted side effects. Additionally, the precorneal residence time of PS in rabbits with DED was 1.8‐fold longer with this new formulation, reducing the number of topical applications required. Above all, this study represents the first DED formulation to only require a once‐a‐day dosing regimen, which greatly enhances patient compliance and therapeutic efficiency.

Luo et al. reported the creation of an epigallocatechin gallate‐loaded carrier that consists of gelatin, thermoresponsive PNIPPAm, and lectin *Helix pomatia* agglutinin.^[25]^ Although PNIPPAm hydrogels have been used extensively in the past, their extreme sensitivity to thermal stimuli greatly limits the number of drug molecules being released. In addition, the poor biodegradability of PINIPPAm hydrogels also constitute another barricade for clinical translation. To overcome these problems, the researchers incorporate lectin, a bioadhesive molecule, to facilitate the contact between the drug‐loaded carrier and the carbohydrates on the mucosal epithelial barrier, thereby prolonging the drug‐cornea contact time. Additionally, the use of gelatin, a degradable matrix, mitigates the issue of biodegradability. Meanwhile, because epigallocatechin gallate, a polyphenol found in green tea, has important antioxidant and anti‐inflammatory properties, the incorporation of it as an active ingredient can diminish inflammation observed in DED patients. Overall, a single instillation of the new eye drop in DED rabbits showed the recovery of deficient corneal epithelium and reduction in oxidative stress and cell apoptosis for 14 days. Essentially, the long‐acting formulation provides an optimistic solution for effectively treating severe ophthalmic conditions via a simple topical application.

#### Glaucoma

Glaucoma is a chronic, progressive ophthalmic disease where elevated pressure inside the eye causes damage to the optic nerve, hence the eventual vision loss.^[49]^ Glaucoma is often divided into two main categories: open angle and angle‐closure.^[49]^ In most cases, a dysfunction in the ocular drainage system leads to a buildup of fluid, ultimately leading to an excessive increase in intraocular pressure (IOP).^[49]^ Presently, eye drops constitute the most common form of glaucoma management.^[50]^ However, penetration into the deeper tissues of the eyes remains a challenge with this method.^[51]^ Moreover, drug loss through nasolacrimal drainage is a frequent issue with conventional formulations, thereby resulting in low ocular bioavailability and high systemic exposure. As glaucoma is often characterized by changes in pH and temperature in the pre‐corneal region, hydrogel‐based eye drops for glaucoma treatment are gaining popularity due to the hydrogel's ability to readily adapt to changes in the environment.

Rawat et al. developed a dual‐responsive gel of nebivolol consisting of ion‐sensitive polymers kappa‐carrageenan and thermoresponsive poloxamers 407 and 188 for the long‐term management of glaucoma.^[27]^ At critical temperatures, P407 undergoes gelation as hydrophobic copolymer chains begin to accumulate to form micelles. Due to the low viscosity of the gel formed by P407 alone, P188 is incorporated to strengthen the overall viscosity of the gel. Meanwhile, carrageenan is a polysaccharide consisting of D‐galactose and D‐anhydro‐galactose disaccharide repeating units. Different grades of carrageenan can be achieved by adjusting the number of sulfate groups added to the disaccharide repeating units. When immersed in monovalent ions, carrageenan displays ion‐sensitive sol‐to‐gel transition. Ultimately, the combination of carrageenan along with P188 and P407 forms a dual‐responsive gel that can penetrate deeper ocular tissues along with great adhesive properties. The gel also exhibited a sturdy hydrogen‐bond structure with enhanced mucoadhesion, hence the great deformation recovery property. Results from the study show that after 24 h of topical administration, 86% of nebivolol was released through the gel. Additionally, compared to conventional nebivolol suspension, greater concentrations of nebivolol were detected in the aqueous humor with the new gel formulation. Essentially, the greater retention of nebivolol in the eye reduces systemic absorption, thereby rendering this new in situ gel a safe therapy for long‐term treatment of glaucoma.

Yang Lai et al. constructed a pilocarpine‐loaded hydrogel consisting of PNIPPAm, gelatin, and glutathione.^[28]^ This study represents the first effort in creating a thermosensitive gelatin‐PNIPPAm hydrogel as a topical treatment of glaucoma and the first to incorporate glutathione into the formulation. As glutathione (GSH) is ubiquitously present in living organisms and mammal tears, addition of GSH can improve the adhesiveness of the gel to the mucous layer of tears via disulfide bond formation. Consequently, prolonged retention of pilocarpine in the rabbit's eye was achieved without any indication of ocular irritation. Moreover, the effects of the gelatin/PNIPPAm/GSH gel on glaucomatous rabbits lasted for 14 days with just a single topical instillation, whereas the formulation without GSH lasted for only 3 days. Given the high antioxidant properties and the long‐acting nature of the gel, this multifunctional formulation could serve as a safe and biocompatible solution for the chronic treatment of glaucoma.

Although past thermogelling systems of chitosan/glycerophosphate (GP) have been developed, commonly encountered challenges include opaqueness and low solubility. In particular, these gels can only dissolve in dilute acidic solutions, which render the clinical application of such a thermogelling system impractical due to the ocular discomfort induced by the acid.^[29]^ Moreover, conventional chitosan‐based thermogel is known to have a slow gelation time.^[29]^ Pakzad et al. addressed these drawbacks by employing GP/N‐(2‐hydroxy‐3‐trimethylammonium) propyl] chitosan chloride (HTCC) hydrogel and sodium hydrogen carbonate.^[29]^ Compared to chitosan, HTTC is a hydrophilic chitosan derivative with greater moisture retention properties, mucoadhesiveness, and antimicrobial activity. Moreover, the transparency of the HTTC/GP hydrogel will not interfere with the patient's vision when used as a topical eye drop. At ambient temperatures, the HTTC/GP formulation retains a liquid form, which converts into a transparent gel upon contact with ocular surface temperatures. The rheological findings of the novel hydrogel demonstrated that the gelling temperature closely matched the ocular temperature found in ocular hypertensive patients. Meanwhile, a reduction in the gelation time of the hydrogel was achieved through incorporating sodium hydrogen carbonate. Above all, in vitro experimentation with the timolol‐loaded hydrogel displayed an exploded release at first followed by a steady release of timolol over a period of 1 week.

#### Uveitis

Chen et al. investigated the use of low‐deacetylated chitosan and β‐glycerophosphate as an Adalimumab carrier.^[31]^ Although Adalimumab confers greater advantages than corticosteroids, systemic treatment or intravitreal injection of the drug carries significant risks such as immunosuppression or retinal detachment.^[52]^ In addition, patients are often reluctant to receive such invasive treatment to the eye, resulting in low treatment efficacy. In the present study, the researchers aimed to provide a non‐invasive therapy that can achieve a high Adalimumab permeation rate. Upon examination, the newly developed hydrogel eye drops significantly increased the permeation rate and effectiveness of Adalimumab in animal models with uveitis. Moreover, the drug‐loaded hydrogel eye drop released a greater concentration of Adalimumab within a limited time period compared to free Adalimumab. Ultimately, the newly formulated hydrogel represents an important stride in the development of highly effective, non‐invasive uveitis treatment.

#### Covid‐19

**FIGURE 6:**
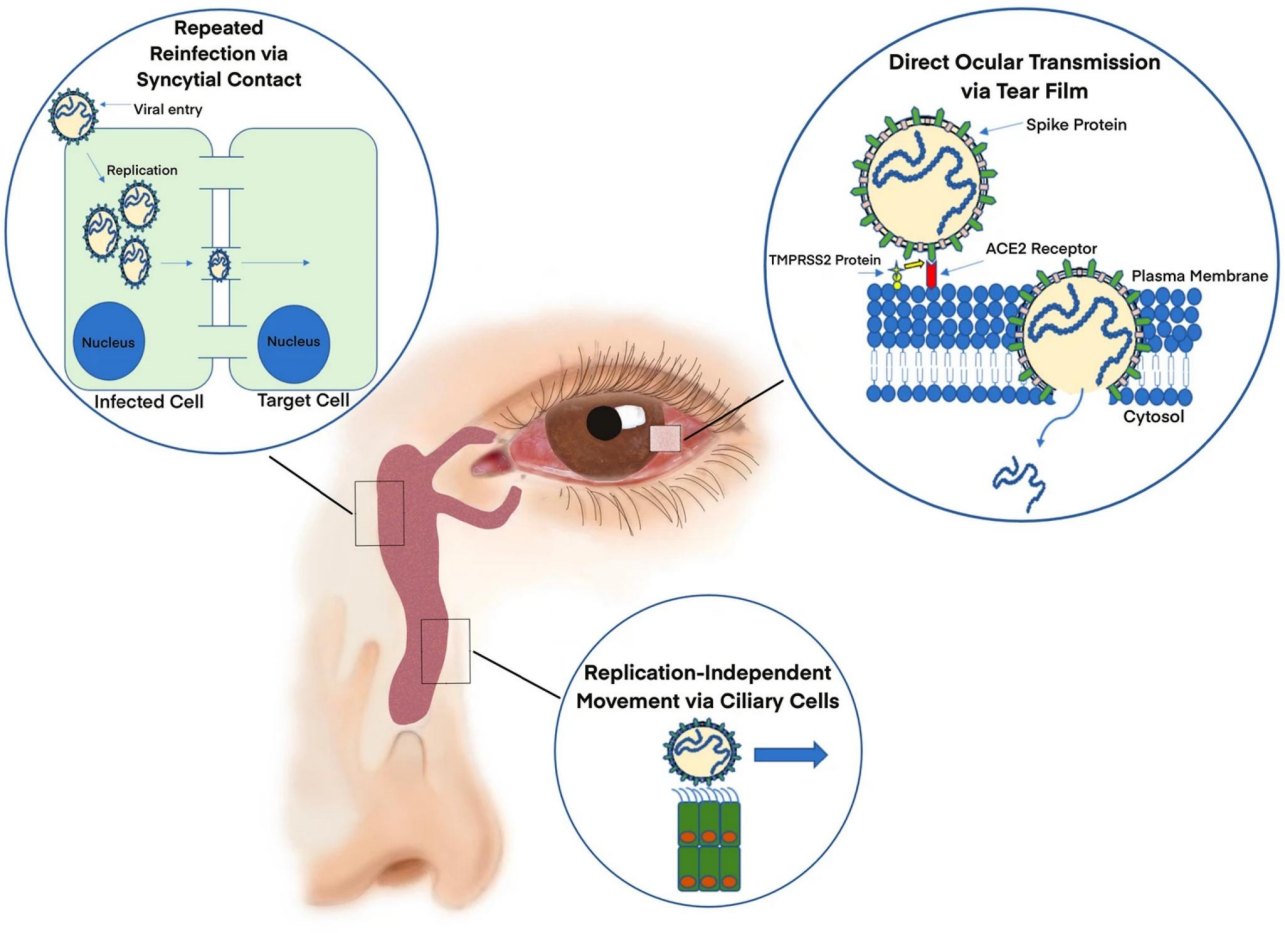
Proposed transmission pathways of SARS‐CoV‐2 through ocular passages. Viruses in infected droplets could escape the antimicrobial activity of the tear film and enter the conjunctiva and cornea before reaching the systemic circulation. On the ocular cell surface, activation of the virus spike protein can be achieved through interactions with TMPRSS2 proteins. Following activation, binding of the spike protein to ACE2 receptors occurs, which permits the virus to enter the host cell. Other potential transmission mechanisms include spreading of the virus from the eye to the lungs. Such transmission could be achieved in two ways: (1) continuous reinfection and replication of neighboring cells that occurs throughout the nasolacrimal duct and (2) replication‐independent travel of the virus that is assisted by ciliary cells of the nasolacrimal duct. Copyright 2022,^[53]^ with permission from Springer Nature.

Transmission of SARS‐Cov‐2 stemming from the eye's anterior segment could potentially lead to exacerbation of respiratory infection (Figure 6).^[53]^ Besides avoiding rubbing of the eyes with contaminated hands, there is currently a lack of protective devices for the eyes against coronavirus.^[54]^ Xu et al. developed the first antiviral ophthalmic drops by using a thermosensitive triblock selenol‐containing hydrogel to deliver Remdesivir (RDV), an FDA‐approved drug for treating coronavirus infection.^[33]^ In this study, the innovative preparation of poly(DHSe/PEG/PPG urethane) hydrogel in low temperatures circumvents the issue of RDV's limited water solubility and instability in high‐temperature conditions. Meanwhile, the addition of selenium serves as an excellent protective agent for the cells residing on the ocular surface. Findings from the study indicated that the polyurethane thermogel was able to retain RDV for 60 min while RDV release in conventional chitosan‐based hydrogel only lasted for 30 min. Compared to the RDV‐loaded cyclodextrin solution, which released all drug content within 1 h, the polyurethane hydrogel eye drop demonstrated a more sustained release of 80% of RDV over a 4‐h period. Fluctuations in IOP 2 h post‐instillation were much more consistent for the polyurethane hydrogel eye drops as compared to those made of poloxamer, carbopol, or PU/HPCD. In the future, more studies should be conducted to confirm if there are additional pathway mechanisms that RDV utilizes besides inhibiting RNA polymerase.

#### Age‐related macular degeneration

Age‐related macular degeneration (AMD) is a vision‐threatening condition that involves the abnormal growth of new blood vessels.^[55]^ The most prevalent therapy for suppressing angiogenesis includes intraocular injection of anti‐VEGF (vascular endothelial growth factors) molecules every 4–6 weeks^[56]^ However, numerous challenges have been raised throughout the years with its use.^[57]^ Anti‐VEGF therapies were shown to induce a persistent increase in IOPs,^[58]^ contribute to the incidence of retinal tears^[59]^ and geographic atrophy progression,^[60]^ induce corneal endothelial and limbal mesenchymal stem cell injuries due to multiple drug administrations,^[61]^ and reduce the choroid vessel thickness.[^62^, ^63^] Furthermore, a recent retrospective study showed that anti‐VEGF intravitreal administration for wet AMD over a 10‐year period still leads to a decline in visual acuity, with persistent retinal fluid in 72% of affected eyes.^[64]^ Furthermore, several limitations are associated with this lifelong, invasive treatment, including elevated risk of retinal detachment, low patient compliance, and high healthcare cost. Given these major drawbacks related to intravitreal administration of anti‐VEGF agents, numerous efforts have been deployed in the past years to develop novel DDS that could leverage these challenges.

To this end, Xin et al. presented the first ophthalmic drop for treating AMD via autophagy of aggregated protein and amyloid beta deposition.^[32]^ The study also exemplified the first formulation to include (3β, 25R)‐spirost‐5‐en‐3‐ol (Dio), a natural, amphiphilic molecule capable of down‐regulating VEGF expression and inhibiting amyloid‐beta deposition. Although Dio possesses considerable benefits over the conventional dexamethasone or prednisolone steroids, its hydrophobicity along with difficulty in traversing biological barriers render the fabrication of Dio‐based eye drops difficult. In this study, nanoparticles filled with Dio molecules were embedded in a calcium alginate hydrogel. The eye drop was then released in pH 7.4 and pH 5.2 blood environments to mimic normal and inflammed (acidic) conditions in native human eyes, respectively. The release of Dio molecules was quicker in inflamed conditions and lasted for 45 minutes while a slower drug release was observed in neutral environments (30 min). Meanwhile, full release of a solution containing free Dio molecules lasted for a mere 5 min in both pH levels. The pH‐sensitive drug release of the hydrogel eye drop allowed for a better control of both drug duration and dosing intervals. Additionally, the encapsulation of Dio molecules in the hydrogel allowed the drug to cross both corneal and blood‐eye barriers, resulting in the buildup of the drug in the vitreous chamber, retina, and choroid. Given the ability of the Dio‐eye drop to effectively suppress neovascularization and cellular migration in AMD primates, the present study constitutes a major progress in the development of noninvasive human AMD therapy.

### 3.2 Contact lenses

Smart hydrogel engineering as contact lenses has seen significant growth over the past decades for the treatment of ocular diseases, as well as a preventive tool. In this section, recent studies regarding these applications are reviewed.

#### IOP monitoring

The gold standard for patient's IOP assessment is the goldmann applanation tonometry (GAT) in a doctor's office.^[65]^ The method possesses several limitations as it not only involves a major time and space restriction but is also unable to provide a continuous recording of the patient's IOP.^[65]^ As IOP can fluctuate due to postural changes or personal circadian rhythm, the one‐time IOP recording of GAT greatly challenges the ability of both patients and clinicians to holistically evaluate IOP trends.^[66]^ Moreover, as GAT is a highly complex optical instrument, only trained professionals are able to provide such measurements. In recent years, smart contact lenses as an alternative for IOP monitoring devices have gained major popularity due to the convenience and accuracy of the lenses provide. Although constant progress has been made on developing contact lens sensors for 24h IOP monitoring, current models often encounter challenges such as the need for a power supply or bulky electronic components causing vision interference or corneal damage.^[67]^

**FIGURE 7:**
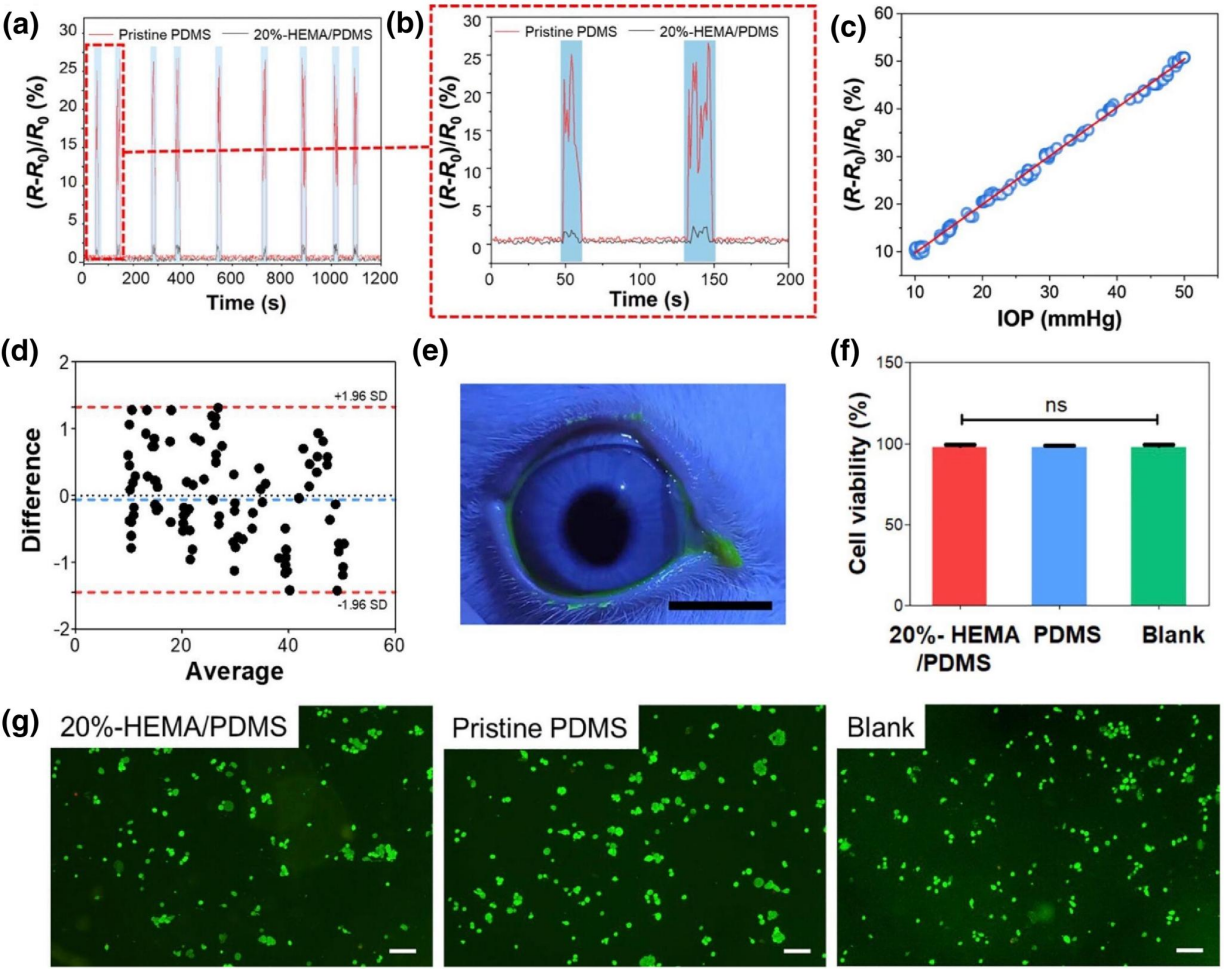
Pristine PDMS based sensor for IOP recording signals in a rabbit eye. (a) Recordings of IOP signals in a rabbit eye using the novel sensor and a sensor made of pristine PDMS. (b) Closed‐up image of 0–200 s. (c) Comparison of the novel IOP sensor and a commercial tonometer. (d) Bland‐Altman plot for comparison of the novel contact lens sensor. (e) Corneal fluorescein after 24 h of contact lens wear showing no corneal epithelial irregularities. Scale bar: 1.5 cm. (f) Viability of cells in 20%‐HEMA/PDMS, pristine PDMS, and blank groups, statistically insignificant, *p* > 0.05. (g) Live (green fluorescent) and dead (red fluorescent) 3T3 cell images at 48 h. Scale bar: 100 μm. Reprinted (adapted) with permission.^[69]^ Copyright 2023, American Chemical Society.

Most traditional noise‐reduction methods involve bulky external circuits, which induce additional noise interference and prevent the sensor from accurately detecting IOP signals.^[68]^ To effectively reduce blinking‐induced noise interference, many previous studies have attempted to reduce the surface coefficient of friction by attaching a lubricant to the contact lens surface.^[69]^ However, this method is rather impractical due to the expensive costs, sophisticated production process, rapid drainage of lubricant, and ocular irritation. Ren et al. (Figure 7) resolved the aforementioned issues by creating contact lens sensors with anti‐jamming ability.^[69]^ Inspired by fish skin, the researchers implemented a self‐lubricating layer, made of HEMA/PDMS hybrid, into the sensor that can reduce the surface coefficient of friction. Consequently, this significantly enhances the sensor's ability to filter out noise interference and exhibits high sensitivity to IOP signals. Overall, the sensor's high biocompatibility along with its ability to record IOP changes represents an important advancement in the creation of wearable and reliable IOP devices.

Ye et al. proposed the innovative creation of a contact lens sensor that changes color while monitoring IOP and matrix metalloproteinase‐9 (MMP‐9), a biomarker found in tears of glaucomatous patients.^[70]^ The study deviates from past works in that it is able to produce a biocompatible smart contact lens sensor that can simultaneously detect two analytes without needing an external power supply. Particularly, an anti‐opal structure and gold nanobowls (AuNBs) are attached to the exterior of the PHEMA‐based contact lens to minimize vision interference. When IOP elevates, the corneal surface bulges outwards, which leads to the deformation of both the contact lens and the antiopal structure, resulting in color changes. When implemented on a porcine eye, the contact lenses change their colors from green to greenish blue as the IOP rises. Meanwhile, MMP‐9 detection was achieved through the cleavage of peptide‐modified AuNBs present on the contact lens surface. Although the dual‐function sensors represent an optimistic alternative, the device may only be suitable for glaucomatous patients without DED. As suboptimal water content in the eye can lead to inaccurate IOP measurement of the device, further studies are needed to create dual‐function sensors that are suitable for glaucomatous patients with DED.

**FIGURE 8:**
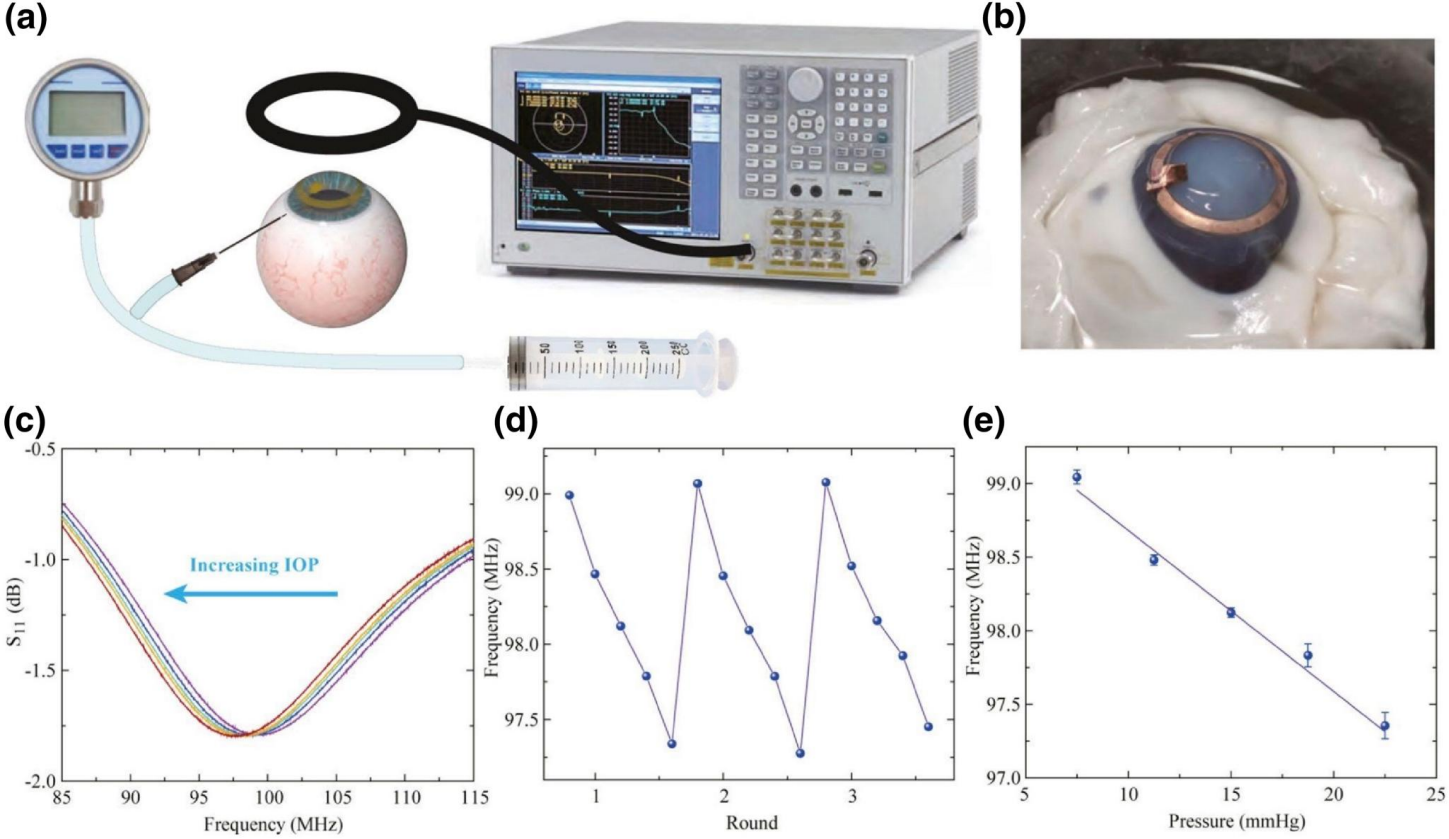
Wireless IOP recording on the in vitro porcine eye. (a) Schema of the IOP measurement device that includes a peristaltic pump, a hydrostatic gauge, and a near‐field coil connected with a vector network analyzer(VNA) for signal readout. (b) Application of a smart contact lens on a porcine eye. Wireless recording of the S_11_ curve (c) and frequency response at various IOPs (d, e). Reprinted (adapted) with permission.^[71]^ Copyright 2022, American Chemical Society.

Similarly, Zhu et al. reported the production of a novel PHEMA‐based contact lens for LCR‐based wireless IOP tracking (Figure 8).^[71]^ Although hydrogel‐based contact lenses are available commercially, difficulty in incorporating stiff electronic components into the contact lenses renders the development of wireless IOP measurement devices challenging.^[71]^ In this study, the radius of the electronic components and the PHEMA hydrogel substrate were designed to precisely match that of the in vivo cornea. Meanwhile, the spherical pyramid shape adopted by the microstructured dielectric elastomer enhances the LCR sensor's sensitivity to IOP signals. Essentially, because all functional components carry a spherical shape, successful integration of the hydrogel and electronic devices into the contact lenses was achieved. Compared to previously produced smart contact lenses, the product presented in this work carries several advantages. First, excellent ocular oxygen and water permeability is obtained using the PHEMA hydrogel, making the smart contact lens safe for long‐term wear. Second, the low cost along with simple and reliable fabrication processes carry significant potential for mass production. Third, the pyramid microstructure was successfully transformed into a spherical shape with minimal deformation.

**FIGURE 9:**
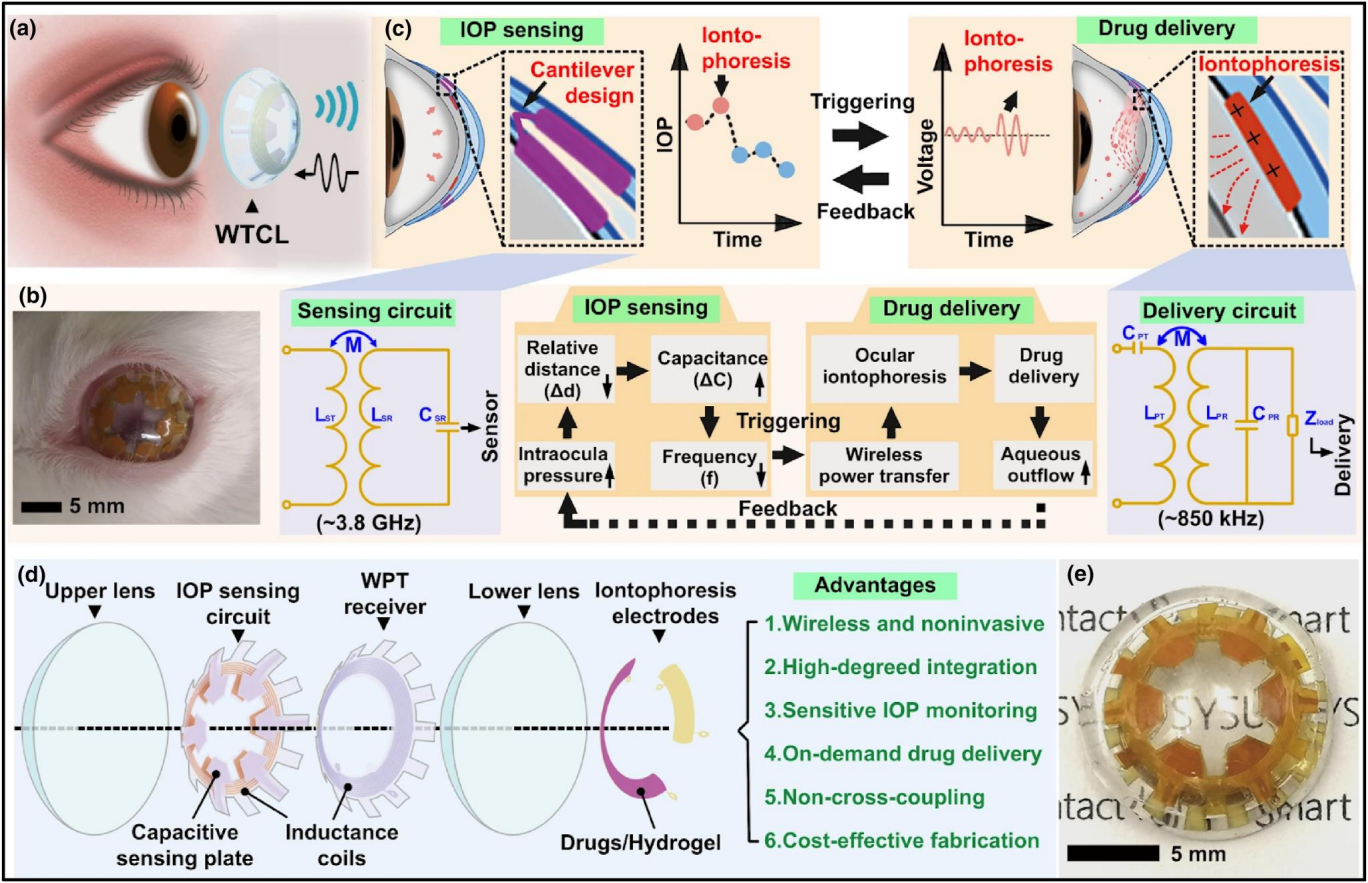
(a) Wireless IOP monitoring and application using wireless theranostic contact lens (WTCL). (b) Image of WTCL applied to the eyes of a live rabbit. (c) Schematic of wireless operation for the purpose of IOP‐sensing and drug delivery. The double‐layer contact lens structure contained an LCR and WPT receiver circuit. An external antenna was attached to these modules, which allowed for the recording of IOP signals and induced iontophoresis for drug administration in times of high IOP. (d) Exploded view of the WTCL structure. (e) Optical image of the WTCL. Copyright 2022,^[72]^ with permission from Springer Nature. http://creativecommons.org/licenses/by/4.0/.

Yang et al. fabricated an LCR‐based theranostic contact lens that not only can detect changes in IOP but can also deliver anti‐glaucoma drug (Brimonidine) in critical IOP conditions (Figure 9).^[72]^ Although theranostic systems have been used previously in medical applications, the restricted dimension of the contact lens renders the application of theranostic systems in ophthalmology difficult.^[73]^ In this work, the integration of both sensing and drug delivering functions is achieved through specialized designs of two unique operational systems. The wireless IOP sensing modulus takes on an LCR circuit configuration, which allows for extreme sensitivity in detecting IOP changes. Meanwhile, the drug delivery modulus contains a wireless power transfer circuit that can induce drug delivery in times of critically high IOP. Particularly, the iontophoretic electrode contains a Brimonidine‐loaded hydrogel layer, which can prompt the delivery of Brimonidine into the aqueous humor through wireless iontophoresis. The use of iontophoresis offers several benefits, including minimal power supply and facilitation of drug permeation across the cornea through electrophoresis effects. In future studies, potential ocular risks associated with extended iontophoresis use should be explored extensively. In particular, the intensity of the current combined with long periods of wear could result in potential ocular inflammation or burn.

#### Glucose monitoring

Although smart contact lenses for monitoring blood glucose levels have been developed, there are various challenges associated with these devices, including opaque electronic components obstructing the wearer's vision, costly signal detection equipment, and the requirement of a power supply.^[74]^ Moreover, many current glucose sensors are dependent upon enzymes to convert glucose into electrochemical signals.^[75]^ This constitutes several limitations for clinical translation as enzymes often have limited lifespan and their function can easily be deformed by changes in the environment.^[75]^ Furthermore, few studies have successfully demonstrated a correlation between blood glucose concentrations and body fluid glucose concentrations. Given that blood glucose can be highly variable according to the glucose metabolism in each patient (*e.g.* pre‐diabetic, diabetic, and non‐diabetic patients), a direct correlation between the glucose concentration according to its compartment of measure cannot be generalized for every patient. For example, blood glucose concentration was shown to be significantly higher in cerebrospinal fluid; however, greater variability was observed in patients with altered glucose metabolism.^[76]^ Using theorical and mathematical models, blood glucose and interstitial glucose concentrations were shown to correlate.^[77]^ However, given the different pharmacokinetics of tears with other body fluids, further studies are required to better explain the possible outcomes of contact lenses as glucose monitoring devices.

**FIGURE 10:**
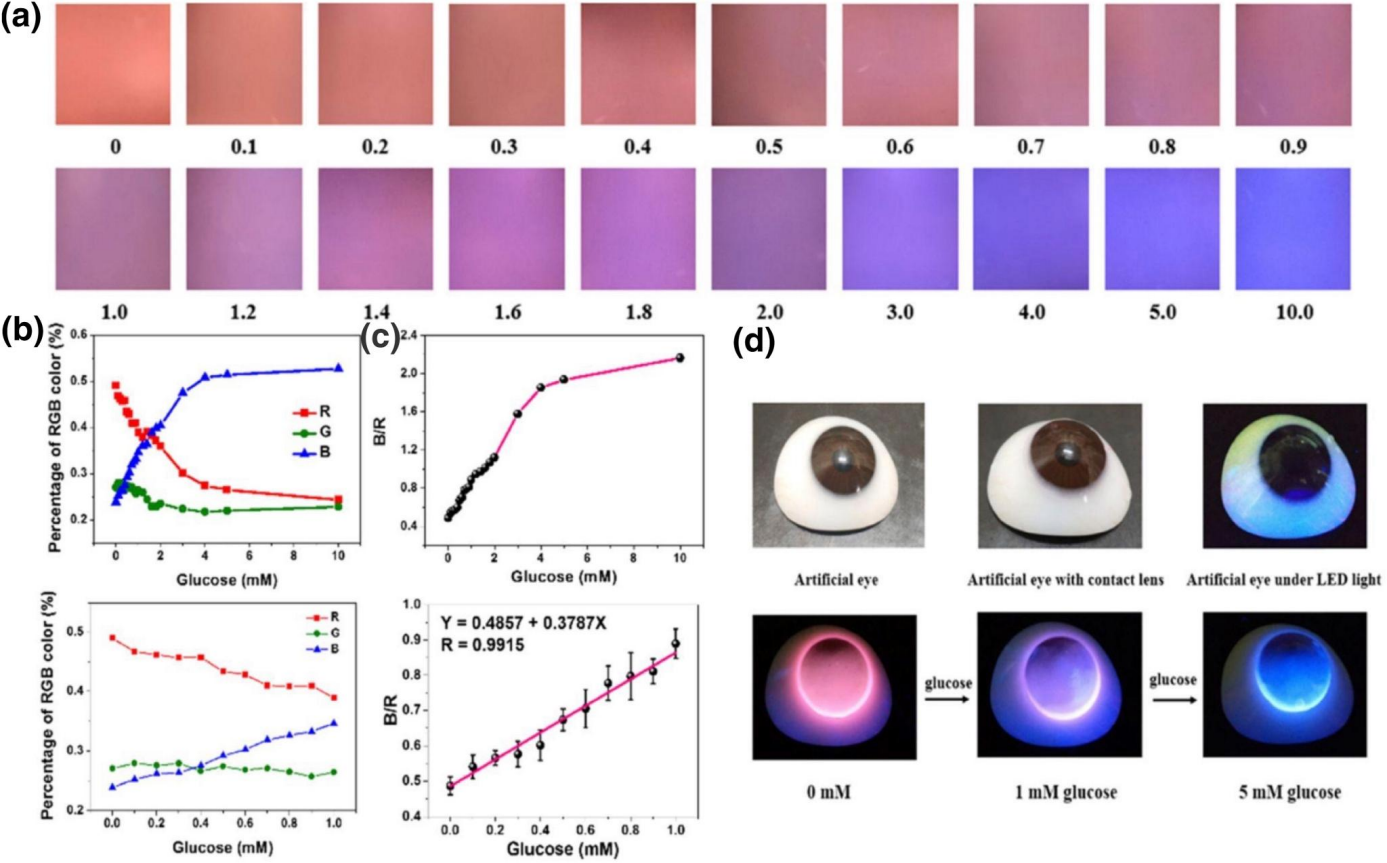
The detection of glucose levels of the smart contact lenses using a smartphone. (a) Fluorescence images of contact lenses in response to different glucose concentrations taken using a smartphone. (b) Normalized % red, green, blue (RGB) figure of contact lenses' fluorescence images at varying glucose levels. (c) Ratios of blue and red channels of the contact lenses fluorescence images at varying glucose levels. (d) Application of smart contact lenses on artificial eyes with varying fluorescent photos of contact lenses in response to artificial tears with different glucose levels. Reprinted with permission.^[78]^ Copyright 2021, Elsevier.

Current fluorescent sensors for contact lenses are still facing numerous challenges, namely insensitivity to low glucose concentrations and incorporation of bulky components. Deng et al. developed fluorescent smart contact lenses that can record minute levels of glucose in tears (Figure 10).^[78]^ Specifically, the contact lens contains a glucose probe and a reference probe that is embedded within the PHEMA hydrogel layer of the contact lens. A rise in glucose concentration prompts the glucose probe to emit an increasingly bright blue light that induces the contact lenses' fluorescent color to switch from red to blue. Wearers can conveniently track their glucose levels through the different colored images displayed on their smartphone. Moreover, the smart contact lenses highly resemble commercially available ones in that they are soft, transparent, and extremely user‐friendly. The glucose probe was found to be capable of absorbing ultraviolet rays, which can prevent UV‐induced ocular damage. Most importantly, glucose detection activity of the contact lens remains stable even when there are other molecules present in the tear fluid. However, there are several limitations that must be addressed before the novel smart contact lens can be widely used. First, because the signal strength and sensitivity of the fluorescence are weak in brightly lit environments, optimal glucose monitoring using the newly developed contact lenses can only occur in dark spaces. Second, as the current stability in glucose detection performance only lasts for a week, the long‐term reliability of the smart contact lens remains unknown. Third, the impact of external temperature on fluorescent signals constitutes a major concern.

Elsherif et al. created bifocal smart contact lenses for continuous monitoring of glucose in eye tears.^[79]^ As myopia is a common complication of diabetes, the innovative contact lenses are capable of providing refractive correction as well as sense changes in glucose concentrations. Particularly, the glucose‐responsive polyacrylamide hydrogel is embedded in the soft contact lens and quantitative measurements of glucose can easily be obtained using a smartphone. The contact lens' curvature provides the focal length for distant vision, while the Fresnel lens (concentric lenses) supplies the near‐vision focal length. Since the Fresnel structure is attached to the hydrogel glucose sensor, glucose elevation in tears would lead to the expansion of the Fresnel structure, thereby leading to a shift in focusing on efficiency and focal length. Compared to previously developed optical sensors, the current model provides significant advantages, including ease of production, rapid responses to glucose changes, easily adjustable detection range, and refractive corrections for myopia and presbyopia.

#### Contact lenses as drug delivery system

##### Antibiotic delivery

Tong et al. suggested the use of polyvinyl alcohol (PVA) nanoparticles to deliver vancomycin in silicone hydrogel contact lenses.^[80]^ Compared to previous methods for consuming vancomycin, the newly developed contact lens not only minimizes systemic exposure but also removes the nuisance of hourly topical applications. Findings from the study indicate that nano‐encapsulation resulted in the gradual release of vancomycin for 48 days. Due to the large size of vancomycin, previous usage of vancomycin is restricted to only Gram‐positive bacteria. Interestingly, the nano‐encapsulated vancomycin demonstrated an unprecedented attack on *Proteus mirabilis* and *Pseudomonas aeruginosa*, which are Gram‐negative bacteria with thick cell walls that are normally unresponsive to vancomycin. Although this study presents a promising solution for effective and sustained delivery of antibiotics, future studies should consider testing this drug delivery system on ocular in vivo models. Additionally, more research must be conducted to understand the consequences of varying certain variables such as vancomycin concentration or storage environment.

Previous attempts at improving bioavailability of econazole, a lipophilic drug, at the corneal surface include loading hydrogels with the drug or use cyclodextrin solutions.^[81]^ However, these methods often come across challenges such as limited permeability across the hydrophilic stroma layer or rapid nasolacrimal drainage.^[81]^ Wong and colleagues proposed a novel method for enhancing econazole topical delivery by combining excess econazole to cyclodextrin solutions and subsequently immersing soft hydrogel contact lenses in the solutions.^[82]^ In this model, cyclodextrin serves to sequester cholesterol present at the corneal surface, thereby improving the permeability of econazole. Results from the study highlighted that the combination of cyclodextrin and hydrogel contact lenses have a 5× more drug released compared to the hydrogel alone. Moreover, almost triple the amount of drug was delivered with the cyclodextrin/contact lens composite system compared to econazole eye drops alone. Most importantly, the composite system demonstrated great therapeutic efficiency against the four fungal species involved in fungal keratitis, indicating great potentiality for treating acute keratitis. Despite the optimistic findings, the econazole minimum inhibitory concentrations (MIC) for the current work is 141 μg/mL, which is overwhelmingly high compared to previously reported MIC values of 8 mg/L for both *Fusarium semitectum* and *Fusarium solani*. The major discrepancy between the two MIC values requires further examination before the loaded contact lenses could be used widely in clinical settings.

##### Tear film improving agent delivery

Akbari et al. designed a nanofiber‐containing ring‐shaped hydrogel that encapsulates HA and is subsequently embedded in a PVA hydrogel‐based contact lens.^[83]^ The incorporation of PVA‐chitosan nanofibers not only improves the contact lens's mechanical durability but also serves as a method for extending the release of HA. Moreover, an increase in the young modulus was achieved with the inclusion of nanofibers, thereby maximizing user's comfortability even after an extended period of usage. The current study also deviates from previous works in that it does not employ the soaking method to load HA, thereby maximizing drug loading efficacy and allowing for a more sustained delivery of HA. Meanwhile, the use of the hydrophobic PVA hydrogel also prevented the adhesion of corneal epithelial cells to the contact lens surface, ultimately reducing the risk of scratching and vision loss.

Previous research has shown that melatonin may be a powerful agent in stimulating tear production.^[84]^ In particular, the ubiquitous presence of membrane melatonin receptors in the eye has prompted many researchers to investigate the efficacy of melatonin and its analog in treating DED.^[85]^ Past evaluations of melatonin analogs on animal models with DED have shown a restoration of tear homeostasis and a reduction in inflammation and oxidative stress. To further confirm this, Navarro‐Gil et al. explored the potential of contact lenses loaded with melatonin analogs in treating DED.^[86]^ Evaluation on rabbits showed a greater tear secretion with the agomelatine/5‐MCA‐NAT‐loaded contact lenses as compared to conventional Diquafosol tetrasodium eye drops. Although melatonin does exhibit significant therapeutic potential, several limitations must be resolved. First, as DED patients often present with a dysfunction in the meibomian gland, future studies are needed to confirm the impact of melatonin analogs on lipid secretion. Second, given the known toxicity of agomelatine on the liver, the toxicity of melatonin and its analog on the eye requires additional investigation. Lastly, although agomelatine has anti‐inflammatory and antioxidative properties, it remains unclear whether the effect is apparent in animals with DED.

##### Intraocular pressure regulating drug delivery

Nikolova et al. proposed a contact lens composed of both poly(sulfobetaine methacrylate) (pSB) and poly(vinyl pyrrolidone) (pVP) as a carrier for timolol maleate.^[87]^ Although previous studies have examined the use of pSB nanoparticles as a drug delivery system for timolol, the application of pSB in hydrogel contact lenses for timolol delivery remains limited. Both pSB and pVP are biocompatible polymers that possess several unique properties of their own. pSB is known for minimizing immune attack while demonstrating high sensitivity to variations in temperature and salt concentration. In particular, pSB has an upper critical solution temperature of around 35–40°C, which could result in effective drug release when it comes into contact with a human ocular surface temperature of 34.5 ± 0.8°C. Additionally, the exposure of pSB polymer to tears, an ionic solution, can result in additional swelling of the pSB hydrogel, thereby enhancing the delivery of timolol. Meanwhile, pVP polymers have numerous applicability in the pharmaceutical industry due to their high oxygen permeability and great mechanical properties. Consequently, the use of pVP polymers as part of the hydrogel contact lens allows for a more comfortable fit of the contact lens around the eyeball. Overall, the combination of different polymers constitutes a rigid physical network that locks the drug inside the hydrogel, thereby extending the releasing time of timolol to 48 h. Compared to conventional soft contact lenses, the copolymeric hydrogel lens presents a unique advantage in that it has a built‐in UV‐B protection capability without requiring further addition of special materials.

Xu and collaborators reported the use of micelles‐laden contact lenses to deliver both latanoprost and timolol.^[88]^ Precisely, the two drugs were loaded in mPEG‐PLA micelles, which were subsequently embedded in the contact lens via HEMA photopolymerization. This study deviates from previously developed nanoparticle‐laden contact lenses in that effective drug delivery is achieved without obstructing the wearer's vision. Testing of the newly developed contact lens in simulated tear fluid indicated a delivery times of 144 and 120 h for timolol and latanoprost, respectively. Meanwhile, the delivery of timolol and latanoprost in the glaucomatous rabbit model lasted for over 168 h. Compared to commercially available eye drops, the novel contact lens has a 79.6‐fold and 122.2‐fold increase in retention time along with a 2.2‐fold and 7.3‐fold increase in bioavailability for both drugs. Despite the optimistic findings, because of the limited oxygen permeability, prolonged usage of the hydrogel‐based contact lens led to corneal edema. Although the issue of corneal edema resolved following contact lens removal, increasing the oxygen permeability of hydrogel‐based contact lenses remains a critical consideration for eventual widespread application.

##### Glucocorticoid drug delivery

Common methods of loading drugs into contact lenses such as drug soaking or drug‐loaded particles often face challenges such as unsustained drug release, limited loading capacity, and inability to load hydrophilic drugs.^[89]^ Because dexamethasone (DEX), a water‐soluble glucocorticoid, is prevalent in treating many ophthalmic diseases, the need to create a sustained delivery system for such drugs remains critical. Inspired by recently developed nanowafers, Liu and colleagues combined KH‐570 into HEMA hydrogel and utilized molecular imprinting technology to create safe and effective DXMS‐loaded SCL.^[90]^ Although past strategies are able to increase oxygen permeability of SCLs through silicone monomers, such addition often leads to ocular irritation.^[90]^ In this work, KH‐570 is supplemented with the hydrophilic HEMA hydrogel to not only enhance biocompatibility but also prevent the SCLs from becoming more hydrophobic. Meanwhile, an increase in drug loading capacity and sustained drug delivery of the SCL is achieved via molecular imprinting technology. However, the use of such technology is frequently accompanied by a deficiency in vision correction. To resolve this matter, the researchers employed an innovative “substitution out of optimization” technique where the optimal composition of SCL was first constructed to achieve optimal comfortability and drug‐delivery function. Afterward, a small monomer in the polymeric network was substituted with imprinting ligands, which allowed the DXMS‐loaded SCL to simultaneously perform both vision correction and ocular drug‐delivery functions. Ultimately, the dual‐function hydrogel contact lens provides great potential for large‐scale clinical applications.

##### Anti‐VEGF drug delivery

Goswami et al. reported the successful synthesis of Avastin‐loaded PHEMA‐based hydrogel lens.^[91]^ Compared to conventional contact lenses, the drug‐loaded hydrogel lens shares a similar surface contour, optimizing wear comfortability. Findings from the study indicate that the accumulation of Avastin between 0 and 1500 min is 60%, with the lens exhibiting 40%–60% anti‐VEGF properties. Although the model constitutes a cost‐effective method for treating diabetic retinopathy (DR), further studies must be conducted on in vivo models to determine the actual safety levels. Additionally, control studies must be implemented to achieve a more accurate understanding of the efficacy of the newly developed contact lens.

##### Statin drug delivery

Previous pilot studies have demonstrated that administration of statins can bring about various ocular health benefits such as hindering the progression of DR or diabetic macular edema (DME).^[92]^ Nonetheless, high oral doses are impractical as they can produce numerous unwanted systemic effects.^[93]^ Presently, scientists are devoted to developing a safe statin ophthalmic formulation that can localize statin effects to the eye without inducing systemic toxicity. In this study, Pereira‐da‐Mota and colleagues proposed the use of an HEMA/EGPEM/APMA‐based contact lens as an ocular delivery system for pravastatin.^[94]^ The hydrogel contact lens presents a unique composition in that it highly mimics the hydrophobic and amino features of the HMG‐CoA binding site. Findings from the study show that the drug delivered by the contact lens demonstrates excellent anti‐inflammatory activity with great permeation throughout the anterior and posterior eye segments. In particular, the accumulation of pravastatin in the cornea, sclera, aqueous humor and vitreous humor was detected after a wear period of 8 h. Moreover, compared to traditional eye drops, much greater bioavailability of pravastatin in the tear fluid was found within 1–7 h of using the new contact lens. Above all, the pravastatin‐loaded contact lens demonstrates similar light transmission and mechanical features to conventional SCL, indicating its potential for comfortable daily wear. In future studies, in vivo tests on human ocular models should be conducted since rabbits often have a lower blinking rate than humans. Moreover, further investigation can be done to evaluate the impact of proteins in the release medium (*e.g.* lysozyme) on in vitro drug release profiles.

### 3.3 Intravitreal and suprachoroidal hydrogel‐based drug delivery

**FIGURE 11:**
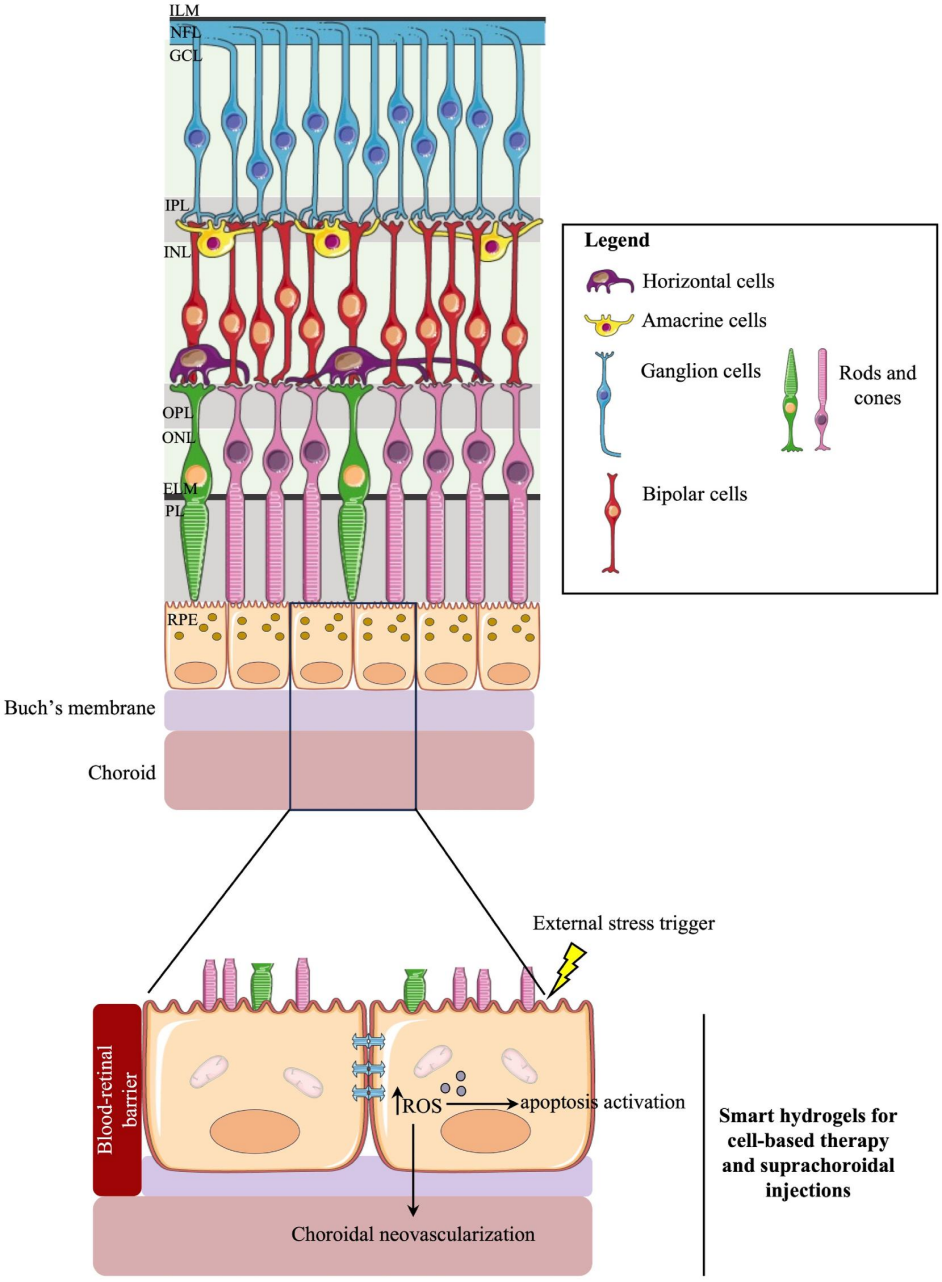
Smart hydrogels for cell‐based therapy in retinal degeneration. The layers (right column) and cell types (left column) of the retina are illustrated. The landmark of retinal diseases is retinal degeneration following apoptosis activation in the retinal pigment epithelium cells, as well as choroidal neovascularization. The use of smart hydrogels for cell‐based therapy and suprachoroidal injections can be a clinical tool to treat retinal diseases. ELM, external limiting membrane; GCL, ganglion cell layer; ILM, internal limiting membrane; INL, inner nuclear layer; IPL, inner plexiform layer; NFL, nerve fiber layer; ONL, outer nuclear layer; OPL, outer plexiform layer; PL, photoreceptor layer; RPE, reinal pigment epithelium. Parts of the figure were drawn using pictures from Servier Medical Art. Servier Medical Art by Servier is licensed under a Creative Commons Attribution 3.0 Unported License (https://creativecommons.org/licenses/by/3.0/).

PSED encompasses a wide range of clinical conditions with genetic heterogenicity and environmental factors, that ultimately lead to the impairment and degeneration of the retinal layer. In the United States, AMD and DR were shown to be the leading causes of retinal diseases (RDs).^[95]^ The pathophysiology involved in RDs has been thoroughly outlined in previous comprehensive literature reviews.[^96^, ^97^, ^98^] Briefly, the microvasculature and anatomy of the retina can be organized into 10 layers, with 6 distinct layer‐specific cell types (Figure 11). The retinal pigment epithelium (RPE) cells are the most superficial cells beneath the choroid and Bruch's membrane, where they possess a crucial role in photoreceptor cell nourishment and homeostasis maintenance.^[99]^ Dysregulation in the RPE cell layer was shown to be a common feature in many RDs.[^98^, ^100^] Once impaired, the non‐functional RPE leads to enhanced levels of oxidative stress modulators with subsequent microenvironment inflammation, neovascularization, autophagy, and apoptosis.

Current treatments for RDs encompass agents that aim to halt disease progression to improve visual function. Since 2004, anti‐VEGF agents have been the backbone treatment of many RDs, such as wet (*i.e.* with neovascularization) AMD and DME,^[101]^ based on the fact that RDs exhibit VEGF‐mediated vascular proliferation and leakage.

Smart hydrogels as advanced DDS have been extensively studied given their highly coveted stimuli‐responsive profile.^[102]^ By modulating the pH, temperature, light and concentration of biomolecules, drug cargo delivery and release, with predictable kinetics, in specific locations is achievable. These systems can highly impact the efficacity and safety of intravitreally administered drugs by decreasing the number of injections needed within the span of a year, promoting biocompatibility and drug efficacy through in situ hydrogel formation, and overcome manufacturing challenges. In this section, the recent smart hydrogel DDS will be reviewed for intravitreal and suprachoroidal applications based on their nature.

Given that PEG‐based hydrogels have been approved by the FDA as drug carriers for the treatment of non‐ophthalmic conditions, numerous efforts have been deployed in the past years for the engineering of intravitreal DDS made from these polymers.[^103^, ^104^] Murakami et al. have demonstrated the ability of tetra‐armed PEG hydrogels for controlled release of Bevacizumab (*i.e.* an anti‐VEGF drug) depending on polymer concentration: higher concentrations of tetra‐PEG gel was linked with optimal hydrogel swelling capabilities in vitro and slower drug release.^[105]^ In a laser‐induced rat model of choroidal neovascularization, phenotypical impacts of the anti‐VEGF aflibercept drug, loaded with microspheres on a thermoresponsive PEG‐*co*‐lactic acid (PLLA) diacrylate (DA)/*N*‐isopropylacrylamide (NIPAM) hydrogel (PEG‐PLLA‐DA/NIPAM), on the retina were observed: lesion area sizes were significantly reduced up to 12 weeks post injection and maintained until 6 months with good biocompatibility and safety.[^106^, ^107^, ^108^] Swelling ratios of hydrogels are known to be proportional to the mesh size of hydrogel.^[109]^ Fine tuning the swelling ratio of hydrogels is achievable through polymer concentration and external temperature modulation, modifications that have been exploited for DDS engineering for controlled drug release though smaller mesh sizes.^[110]^ Additional thermoresponsive hydrogels have shown their efficacity for anti‐VEGF drug delivery based on this principle, such as the PEG‐block‐poly(lactic‐co‐glycolic acid) (PEG‐PLGA‐BOX) hydrogel^[111]^ and the PEG‐poly(propylene glycol) (PPG), and poly(ε‐caprolactone) (PCL) hydrogel.^[112]^ Although no exact treatment regimen exists for anti‐VEGF administration, a recent systematic review suggested that over the first year of treatment, monthly regimens were more effective than PRN regimens (*i.e.* 7–8 injections in a year).^[113]^ These clinical outcomes can potentially be attributed to the decrease in intravitreal drug concentrations, challenges that can be leveraged with DDS using smart hydrogels.

Alongside the development of DDS for anti‐VEGF agents, efforts have also been deployed to apply hydrogel‐based DDS to miscellaneous intravitreal medications. A triblock copolymer, consisting of NIPAM, *N*‐acryloxysuccinimide (NAS), and PEG, crosslinked with methacrylated DEX in situ at physiological temperatures, has shown that minimal quantities of the hydrogel (*i.e.* 100 mg) were sufficient to induce drug release in the vitreous for nearly 500 days, while maintaining therapeutic levels.^[114]^ The PLGA‐PEG‐PLGA copolymer thermoresponsive hydrogels for DEX delivery were shown to display sol and rheological behaviors: hydrogel gelation is maximal at physiological temperatures and maintained for up to 3 weeks^[115]^ The use of this smart hydrogel was also investigated for the intravitreal administration of ganciclovir (GCV), showing a controlled release pattern of GCV over 96 h,^[116]^ as well as for dual drug delivery of diclofenac (*i.e.* a NSAID) and Bevacizumab.^[117]^ Other successful DDS (*i.e.* non‐biodegradable and biocompatible) were also engineered for the controlled intravitreal release of vancomycin using a polyNIPAAm hydrogel crosslinked with the PEG‐DA polymer.^[118]^ However, drug kinetic remains to be improved: intravitreal administration of vancomycin‐loaded PEG‐based hydrogel resulted in a rapid peak in its concentration in comparison to the topically administered drug, in detriment of a sustainable drug concentration over time.^[118]^

A major challenge for the clinical translation of PEG‐based thermoresponsive hydrogels resides in their prolonged gelation times.^[119]^ To leverage this drawback, using Diels‐Alder reactions, Gregoritza et al. engineered two in situ forming four‐armed and eight‐armed thermoresponsive Poloxamine hydrogels with optimal LCST.^[120]^ These macromonomers were shown to have great stability for up to 329 days (*i.e.* stable viscoelastic hydrogels were obtained at physiological temperature) and they were able to achieve sustained release of Bevacizumab over a period of 115 days at the longest.^[120]^ However, greater cytotoxicity of 4 armed‐Poloxamine hydrogels was observed using in vitro mouse fibroblasts.^[120]^ Similarly, additional Diels‐Alder crosslinked thermoresponsive hydrogels have been developed for the purpose of sustained DDS and demonstrated great potential, such as tetrazine‐modified HA (HA‐Tz) and PEG‐bisnorbornene (PEG‐bisNB) hydrogels.^[121]^

However, given the structural anatomy of the vitreous, intravitreal injections have limits independent of the DDS's nature, which may be leveraged by an alternative route of administration: the suprachoroidal space (SCS)—a region between the sclera and the choroid. Suprachoroidal injections allow to by‐pass the anterior segment, therefore avoiding the risk of intravitreal injection‐induced cataracts and IOP elevation.^[122]^ Furthermore, drug kinetics is influenced in a positive manner: higher levels of medication can reach the posterior segment due to low levels of drug diffusion resistance.[^122^, ^123^] Suprachoroidal injections are performed using microneedles through the sclera, therefore classified as a minimally invasive procedure. Therefore, by combining the SCS route of administration with the innovative method of drug delivery based on smart hydrogels, drug efficacy and safety can be enhanced for many RD's.

The hydrogel swelling capacity of HA‐based smart hydrogels is an effective and safe method for DDS into the SCS. Jung et al. have shown that SCS injection of a double layered HA formulation *in* ex vivo rabbit eyes can instantly deliver over 70% of the drug 3–6 mm into the SCS from the limbus.^[124]^ Using heating the eyes at physiological temperature, hydrogel swelling was shown to induce a pushing effect, therefore significantly enhancing the particle distribution posteriorly (*i.e.* at 9 mm from the limbus into the SCS) and allowing the reach of the optic nerve.^[124]^ The pushing effect can also be positively modulated with increased osmotic flow. However, HA‐based hydrogel administration into the SCS was shown to be associated with a transient decrease in IOP to approximately 4 mmHg for 6 days^[124]^ More recently, the same team have demonstrated the successful sustained in vivo delivery of an anti‐VEGF drug into the SCS of rabbits by engineering a HA hydrogel crosslinked with poly(ethylene glycol) diacrylate (PEGDA) polymer.^[125]^ One hour post injection, the HA‐PEGDA hydrogel went through in situ crosslinking with Bevacizumab and was shown to undergo slow release for over 6 months without any reported ocular damages.^[125]^ The transient decrease in IOP was shown to recuperate gradually over the course of 3 months^[125]^ Conversely, high IOP in glaucoma has been the subject of interest for the development of smart hydrogels. Hao et al. have engineered an in situ forming polyzwitterionic polycarboxybetaine (PCB‐OAA) hydrogel, where its injection into the anterior SCS was shown to enhance aqueous humor drainage and subsequently decreased IOP.^[126]^ These results are attributable to the mainstay properties of smart hydrogels; the expansion of the PCB‐OAA hydrogel within the anterior SCS leads to the expansion of the SCS and subsequent outflow pathway enlargement. Other smart hydrogels have shown promising results for the treatment of glaucoma based on this mechanism.[^127^, ^128^] HA‐PEGDA smart hydrogel was shown to significantly lower IOP levels for 4 months in an in vivo model who received suprachoroidal injection.^[127]^

Overall, smart hydrogels have proven their potential efficiency and safety for the treatment of PSED throughout these preclinical studies. However, interspecies variability must be considered for future clinical translation of thermoresponsive hydrogels. Disparate results were outlined regarding the transient adverse effect of smart hydrogels on IOP: IOP reduce was shown to be lower in monkeys versus rabbits.^[128]^ Given the complexity of the human eye, these results cannot be fully generalized. Furthermore, variability in physicochemical properties within thermoresponsive hydrogels was shown to be dependent on hydrogel modification with polymers.^[118]^ Therefore, a balance between degradability and efficacity must be considered for reaching optimal and sustainable drug concentrations. It is evident that further studies are required to explore the long‐term efficacy, safety, biocomposition and biocompatibility of smart hydrogels for intravitreal and suprachoroidal injections.

### 3.4 Cell‐based therapy delivery using smart hydrogels

Cell‐based therapies, also known as stem cell therapy, are considered the mainstay of regenerative medicine and their applications in the field of ophthalmology have seen a colossal growth over the past decades.[^129^, ^130^] Stem cell therapy can be employed for ASED and PSED. Cell death by apoptosis is a landmark in these ophthalmic conditions. Therefore, stem cell therapy consists of transferring autologous or allogenic cellular material with the intention of inducing cellular regeneration.^[131]^ Although not approved by the FDA for ophthalmic conditions, preclinical and clinical studies have sought to elucidate the delivery of stem cells to the anterior and posterior segment of the eye.^[132]^ The most studied routes of administration of stem cells are the intravitreal site and transplantation methods. However, serious vision threatening adverse effects were reported in patients enrolled in clinical trials for intravitreal injection of stem cells, such as scar formation, retinal detachment, and vision loss.^[133]^ Furthermore, the survival and differentiation of the transplant is worrisome: only a fraction of injected retinal progenitor cells (RPC) reach survival and can further participate in cell regeneration.^[134]^ Using biodegradable scaffolds, it is possible to enhance cell suspension survival following transplantation.^[135]^ Efforts have thus been deployed to expand stem cell delivery methods, mainly through use of hydrogels.^[136]^ In this section, we will review the most recent advances of thermoresponsive hydrogels applied to cell‐based therapies.

The most common sources of stem cells used for the treatment of eye diseases are hematopoietic stem cells (HSC), mesenchymal stem cells (MSC) and human induced pluripotent stem cells.^[137]^ The combination of these stem cells with 3D models of macroporous smart hydrogels have shown promising results in enhancing treatment efficacy. Macroporous hydrogels simulate the spongious architecture of bone marrow, therefore providing an optimal microenvironment for stem cells. Rödling et al. engineered PEG‐DA hydrogels colonized with MSC et HSC: in vitro hydrogel swelling allowed cell seeding.^[138]^ The biomimetic 3D model allowed effective maintenance of HSC in comparison to static culture conditions.^[138]^

Park et al. have demonstrated the use of in situ crosslinked gelatin‐hydroxyphenyl propionic acid (Gtn‐HPA)‐based hydrogel with RPC.^[139]^ Cell density was shown to be a limiting factor for cell viability: higher RPC density within the Gtn‐HPA smart hydrogel showed cell viability levels of over 60% up to 7 days^[139]^ However, with optimal crosslinking conditions, high cell viability levels were obtained. Once transplanted into the eyes of a rat model, RPCs were observed in the subretinal space, with a few cases of retinal detachment.^[139]^ Improper localization of stem cells can lead to potential complications such as subretinal bleb formation and subsequent retinal detachment. Further studies are thus required to investigate the etiologies involved in subretinal hydrogel leakage, as well as to establish solutions, such as enhancing the in situ cross‐linking strength and time. Conversely, Gtn‐HPA polymer hydrogels were crosslinked with HA polymers to form an interpenetrating polymer network, loaded with human retinal ganglion cells (RGCs), and were subsequently injection into the vitreous of a rat model.^[140]^ Gel point was shown to be tunable, ranging from 151 to 167 s based on the concentration of Gtn‐HPA polymers.^[140]^ RGCs showed great viability (>95%) and successful attachment to the inner limiting membrane of the retina.^[140]^ GG‐based smart hydrogels have also shown promising results for retinal cell regeneration.[^141^, ^142^, ^143^] Youn et al. have shown that GG/HA hydrogels exhibit faster stress relaxation due to their viscoelastic properties, a crucial parameter that promotes cell proliferation and differentiation.^[143]^

### 3.5 Hydrogel‐based ocular adhesives for wound repair

Eye injuries are key contributors to the etiologies of blindness and vision impairment. A meta‐analysis estimated that in the United States, 24 million people suffered from an eye injury, of whom over 3.2 million suffered from vision impairment or blindness.^[144]^ It is thus clear that proper management and treatment of eye injuries is crucial. Most commonly, eye injuries involve the cornea. The cornea is composed of 5 main layers, each possessing a crucial role in the maintenance of cellular and tissue homeostasis (Figure 3). Corneal epithelium regeneration is mainly achievable through the action of limbal epithelial stem cells, where its pathophysiology has been thoroughly reviewed by Ruan and his colleagues.^[145]^ Ocular adhesives are the essence of non‐invasive wound repairing techniques related to eye injuries. Not only do they address the shortcomings of sutures, but they also provide functionality to enhance tissue repairing and regeneration through complex mechanisms of action involving molecular interaction.^[146]^ Amongst the pitfall's associate with microsurgical repairing of ocular injuries with suturing, post‐operative astigmatism, inflammation, and neovascularization are feared.^[147]^ Microsurgical suturing also requires a highly skilled surgeon and is therefore not sought as the best treatment method. Conversely, in addition to overcoming these drawbacks, natural ocular adhesives are cost efficient, biodegradable, biocompatible and do not require years of experience and skill sets.

Tissue adhesives encompass synthetic or natural compounds with roles involved in the reconstruction and regeneration of wounds. Cyanoacrylates and their derivatives are amongst the oldest and most common synthetic glues used in ophthalmology. However, their use is yet to be approved by the FDA. The off‐label applications of cyanoacrylate‐based glues (*i.e.* Histoacryl^®^, Nexacryl^®^, Dermabond^®^) involves a wide range of clinical conditions, such as corneal perforations, neurotrophic or chemical keratitis, corneal melts, and bleb leaks following glaucoma surgery.^[148]^ They have shown to be efficient in their ability to halt keratolysis progression with added antibacterial activity.^[149]^ Although, a major concern regards their cytotoxicity to the corneal endothelium and intraocular lens.^[150]^ They were shown to induce acute inflammation and infection complications, as well as precipitate cataracts, processes that could inevitably aggravate the underlying eye condition. Furthermore, for perforations larger than 3 mm, cyanoacrylate‐based glue use comes with its challenges: leakage into the anterior chamber through the perforation hole could lead to the development of cataracts, glaucoma, or granulomatous keratitis.^[148]^ Therefore, in these specific cases, suturing may be inevitable. Fibrin adhesives (*i.e.* Evicel^®^, Tisseel^®^, Artiss^®^) were shown to be a great substitute to synthetic glues such as cyanoacrylate glues, given their biological nature, hence their biocompatibility.^[151]^ However, fibrin glue is formulated through fibrinogen extraction from donors, thus providing a major risk of blood‐borne disease transmission.^[152]^

Hydrogel‐based ocular sealants have gained interest in the past years due to their highly biocompatible nature, low toxicity, and absence of disease transmission. Successful engineering of hydrogels as adhesives has to meet specific requirements such as high leak pressures to withstand IOP, rapid cross‐linking, refractive index similar to the corneal index, and mechanical properties allowing efficient sealant function (*i.e.* swelling capabilities, viscosity, high diffusion coefficient, strong adhesion strength, optimal degradation time).^[147]^ Copolymerization of the hydrogel subtypes (*i.e.* synthetic, natural, or semi‐synthetic) using diverse platforms and technologies, such as the stimulus responsiveness of biomaterials, has allowed to further the development of ocular adhesives. In 2014, the FDA approved its first synthetic polyethylene glycol (PEG)‐based hydrogel ocular sealant (*i.e.* ReSure^®^) for clear corneal incisions in cataract removal and IOL implantation surgeries. In parallel, OcuSeal^®^, a dendrimer‐based synthetic ocular adhesive, was approved in Europe.^[153]^ Since then, multiple studies have demonstrated the efficacity of ReSure^®^ in wound repair and corneal epithelialization, such as in post LASIK surgery for buttonhole formation recurrence prevention^[154]^ and pars plana vitrectomy wound closure.^[155]^ However, major challenges exist with the use of ReSure^®^. For example, the ocular sealant must be applied within the 14–17 s following mixing due to its rapid gelation time, it cannot be applied on actively leaking wounds, it is unstable on the ocular surface after a period of 3 days, it cannot fill‐in the volume of stromal defects, and requires a tissue to prevent its fall.^[156]^ Given these limitations, numerous studies have investigated the use of other hydrogel adhesives for ocular wound repair, a clinical condition yet to be approved by the FDA for ocular hydrogel‐based adhesives. In this section, studies within the last 5 years are discussed.

Stimuli‐responsiveness is a property of hydrogels that has been exploited in preclinical studies for ocular wound repair. The ability to tailor their behavior and mechanical properties to shape‐fill ocular wounds upon injection is a primordial advantage because most eye injuries possess irregular margins and deficit variability. Furthermore, thermoresponsive (*i.e. smart*) sealants (TRS) can easily be removed with cold water,^[157]^ giving more flexibility for the actual procedure and subsequent treatments without damaging the surrounding corneal tissue. The biomolecular mechanisms involved in the role of smart hydrogels as ocular adhesives have thoroughly been reviewed by Grinstaff.^[158]^ To achieve their function as biomolecular adhesives, smart hydrogels require a large number of crosslinked polymers as the backbone. Once applied on the ocular surface as a solution, the formation of a highly complex polymer with the biological tissue is achieved through photo‐crosslinking, nucleophile‐electrophile crosslinking, or peptide ligation.^[158]^ These 3D molecular changes successfully seal the lacerated ocular tissue.

Poly(*N*‐isopropylacrylamide) (PNIPAM), a thermoresponsive polymer, was studied as an adhesive for the closure of sclerotomies, results yielding excellent wound closures in a rabbit model.^[159]^ Furthermore, copolymerization of PNIPAM with *N*‐*tert*‐butylacrylamide (NT) or butylacrylate (BA)—synthetic monomers known to decrease the LCST of PNIPAM and enhance its mechanical properties^[160]^—was performed to engineer smart hydrogels with gelation points of 22°C and 16°C respectively.^[161]^ Using an ex vivo cadaveric porcine eye model with globe rupture, PNIPAM‐BA was shown to have more adhesion power; PNIPAM‐BA could maintain ocular pressures above 70 mmHg without any leakage, whereas PNIPAM‐NT was shown to withstand pressures to up to 40 mmHg.^[161]^ Furthermore, in rabbits with 3 mm full thickness lacerations of the sclera repaired with TRS, it was shown at the 4‐week mark that these hydrogels form a barrier capable of occluding the globe rupture for 72 h, while waiting for subsequent treatment.^[161]^ However, the occlusion is not complete, IOP values reaching 60% of the expected values.

**FIGURE 12:**
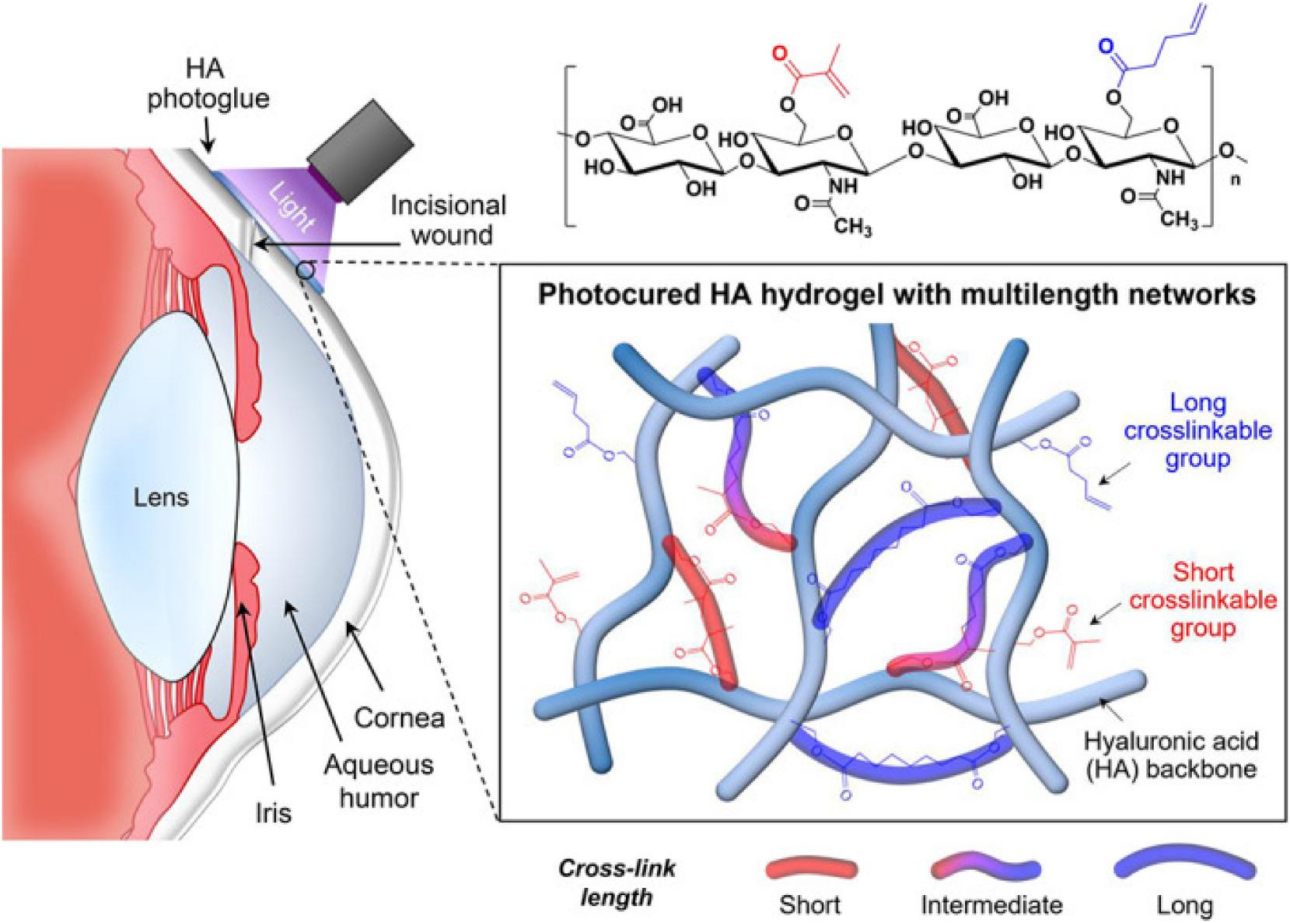
The schematic illustration of hydrogel‐forming ocular glues with cornea‐like optical transparency for ocular wound repair. Following light exposure, the HA‐based photocurable glue forms a hydrogel barrier on the wound site. Copyright 2022,^[171]^ with permission from Bioengineering & Translational Medicine (http://creativecommons.org/licenses/by/4.0/).

Natural hydrogels, such as gelatin and HA hydrogels, in combination with crosslinkers have been sought as potential ocular adhesives given their well‐established biocompatibility. Methacrylated hydroxyl dendrimer (D‐MA) photocrosslinking with methacrylated HA, also known as the OcuPair^TM^ hydrogel sealant, was shown to stabilize different full‐thickness ocular wounds—with linear incisions up to 6 mm and stellate incisions of 3.5 mm—in rabbits and withstand IOPs of 70 mmHg and above in ex vivo porcine eyes.[^162^, ^163^] Ocular adhesion of the OcuPair^TM^ sealant persisted for up to 5 days with excellent biocomptability; no signs of corneal inflammation were observed.[^162^, ^163^] Furthermore, an adhesive patch composed of 3 photocrosslinkable polymers (*i.e.* methacrylate (MA)‐functionalized gelatin (GelMA), HA glycidyl methacrylate and PEG diacrylate) was shown to possess higher adhesive properties in comparison to the ReSure^®^ sealant, with successful occlusion of 4 mm epithelial defects.^[164]^ Similarly, combined oxidized HA and GelMA hydrogel (GMO bioadhesive) use in a rabbit conjunctival defect model was shown to successfully adhere to the 8 mm defect, however partial degradation was observed on day 14.^[165]^ Nonetheless, epithelial regeneration was observed on the edges of the scaffold.^[165]^ Modulation of the GelMA and HA ratios in an ECM‐like hydrogel were shown to enhance cell viability through the modulation of corneal microenvironment.^[166]^ More recently, Qian and colleagues have developed a highly transparent bioadhesive, with higher adhesion power, biocompatibility, and regenerative properties to meet the needs in ophthalmology.^[167]^ *In situ* oxidative free‐radical polymerization of GelMA with dopamine methacrylamide was shown to enhance corneal epithelial cell proliferation, while preventing proinflammatory microenvironment by decreasing ROS accumulation.^[167]^ GELGYM, the precursor of Gelatin‐glycidyl‐methacrylate hydrogel with twice the potency of GelMA to hold methacrylate groups, has also showed promising results as an ocular adhesive with in vitro and ex vivo biocompatibility.^[168]^ GELGYM confers greater safety in comparison to GelMA given the possibility to photocrosslink at lower intensities. Another photocrosslinkable ocular adhesive is the GelCORE polymer. Shirzaei Sani et al. have shown that GelCORE—a bioadhesive hydrogel made of gelatin and photoinitiators (PIs)—mimicked the properties of the native cornea with great physiochemical properties.^[169]^ In fact, GelCORE possesses tunable elastic moduli and in vitro enzymatic degradation levels influenced by external stimuli. The ultimate tensile strength (UTS) and elastic moduli of GelCORE were shown to significantly increase with prolonged photocrosslinking times.^[169]^ In spite of these results, photocrosslinking is known to require a prolonged period of light exposure and high concentrations of radical‐generating PIs,^[170]^ opening the door to potential eye cytotoxicity's. To leverage these challenges, a group has proposed the use of multilength‐networked HA hydrogel (HA photoglue), based on short and long photocrosslinkable groups (Figure 12).^[171]^ HA photoglue was shown to instantly activate its adhesive properties due to rapid photocuring, with excellent transparency (light transmittance was over 95%) and successful uniform epithelial regeneration.^[171]^ Furthermore, crosslinking of HA with host groups—cucurbit[n]urils (CB[n]) and cyclodextrins (CD)—are known as supramolecular HA (s‐HA) hydrogels and possess shear‐thinning and self‐healing behaviors due to spatiotemporal interactions.^[172]^ It was shown that CECs and MSC encapsulation in s‐HA hydrogels enabled stem cell adhesion to corneal stroma at day 4 post treatment, subsequent secretion of growth factors, chemokines, and cytokines for MSCs proliferation, and wound healing after in vivo eye injury.^[173]^

Chitosan‐gelatin (CHI) smart hydrogels, individually and in combination with other platforms, have also been studied to engineer successful ocular adhesives. Exogenous recombinant stromal cell derived factor‐1 (SDF‐1) was shown to enhance in vitro LESC and MSC chemotaxis and proliferation through the upregulation of SIX homeobox 1 (SIX1), microphthalmia‐associated transcription factor (MITF), epidermal growth factor, and hepatocyte growth factor mRNA expression.^[174]^ SDF‐1 in combination with the thermosensitive CHI hydrogel—possessing a gelation temperature of 37°C—promoted nearly complete corneal repair in an alkali‐injured rat model through enhanced ΔNp63 positive‐cell ratio, with lesser neovascularization levels, when compared to control groups.^[174]^

Although synthetic hydrogel polymers allow customization, tissue integration and regeneration is inferior given the use of chemical crosslinkers.^[175]^ Conversely, natural polymers were shown to induce corneal thinning and fibrosis in individuals.[^176^, ^177^] Therefore, to overcome these drawbacks, recent studies have focused on the development of human decellularized corneal matrix hydrogels (hDCMH) as corneal sealants to prevent trauma‐induced scarring and allow stromal regeneration.[^178^, ^179^] hDCMH are innovative given that they harbor major ECM components in a similar way to the native human cornea; approximately 74% sulfated glycosaminoglycans and 50% collagen were shown to be retained in hDCMH in comparison to native corneal tissue.^[178]^ Furthermore, hDCMH were shown to be thermosensitive, with gelation capabilities at physiological temperature and pH.^[178]^ *In vivo* experiments by the same group have shown the safety of hDCMH given the absence of pyrogenic and immunogenic reactions. In a rabbit corneal injury model, with epithelial defects up to 3 mm in diameter and 200 μm in dept, it was shown that wound filling with hDCMH significantly reduced the size of the epithelial defect as soon as 7 days post application, and corneal thickness was similar to those of native corneas by 90 days post‐surgery.^[178]^ Similar results were obtained by incorporating hDCMH microparticles into human fibrin sealant.^[179]^

Overall, hydrogels for ocular wound repair present numerous advantages compared to the conventional synthetic glues and fibrin‐based natural sealants. Given their stimuli‐responsive property and their tunable mechanical properties through copolymer crosslinking and photocrosslinking, they are biocompatible and exhibit great potential for complex eye injury healing. The possibility to modulate the level of UTS, size of surface area and dept, all by maintaining the native cornea properties are clinically captivating properties of hydrogel‐based ocular adhesives that improve personalized medicine. However, limitations exist and need to be leveraged. Studies regarding the prolonged usage of hydrogel‐based ocular adhesives are limited; thermosensitive hydrogels have shown great potential in acute phases of injuries. Their toxicity in the long term is yet to be elicited. Furthermore, their use has been proven in specific full‐thickness injuries, limiting their use to smaller injuries. In cases of significant globe ruptures, conventional treatment is still sought, and hydrogels cannot be the sole treatment. Further investigations regarding their safety and efficacity in the long term are required, as well as their applications to complex eye injuries.

### 3.6 Hydrogel‐based vitreous substitutes

The vitreous body is a transparent gel‐like structure that has tremendous importance in biochemical and physiological functions, as well as in the optical properties of the eye. Numerous vitreoretinal diseases can lead to the substitution of the vitreous body with another solution, a process known as vitrectomy, as part of the medical treatment.^[180]^ The physiological vitreous is mainly composed of water, representing 98% of its composition, as well as collagen, and glycosaminoglycans (*i.e.* HA, chondroitin sulfate, and heparan sulfate).^[181]^ The main challenge regarding vitrectomy is that no currently approved vitreous substitute has a perfect resemblance to the native vitreous. The ideal vitreous substitute should meet specific requirements.^[181]^ The viscoelastic properties should be like those of the native vitreous, to maintain and withstand normal IOP. Furthermore, the substitute should be optically transparent, easy to manipulate, durable and non‐biodegradable, biocompatible, non‐toxic, and with low liquefaction thresholds. Current vitreous substitutes can be divided into two main categories: gases or liquids. Sulfur hexafluoride (SF_6_) and perfluoropropane (C_3_F_8_) are the most used gases in the treatment of vitreoretinal diseases, compared to air and hexafluoroethane (C_2_F_6_). However, these substances can lead to gas‐induced cataract formation and corneal endothelial changes.^[182]^ Furthermore, postoperative care may be challenging in certain patients with limited mobility, given the fact that a prone positioning is required as soon as possible. Conversely, liquid substitute (*i.e.* Perfluorocarbon liquid, semifluorinated alkanes, silicone oil (SO), and heavy silicone oil) have shown great potential as vitreous substitutes, however their use was shown to be associated with possible complications.[^181^, ^182^] Given the drawbacks associated with the current gas and liquid vitreous substitutes, the use of polymers as vitreous substitutes has been investigated given their capabilities into meeting all criteria's.

Smart hydrogels have shown promising results in preclinical studies as for fulfilling the mentioned criteria of ideal vitreous substitutes. Given their synthetic polymer network, they have the capacity to expand their volume by water.^[181]^ By externally modulating the pH, temperature, light, pressure, and chemical composition, smart hydrogels can transform into a 3‐dimensional structure.^[183]^ These properties of smart hydrogels facilitate their delivery, given their rapid gelation under physiological conditions. Moreover, smart hydrogels having the ability to easily mold the defect, it renders their use less invasive and more accessible for complex vitreoretinal diseases.^[184]^ In addition, their high‐water content allows them to leverage the refractive index issues related to other vitreous substitutes, such as gases and SO. However, as of today, no smart hydrogels have yet been approved given the observed shortcomings in previous studies.[^185^, ^186^] In this section, we will review the most recent studies regarding smart hydrogel use as a potential treatment for vitreoretinal diseases. Previous studies (>5 years) have also reported the use of non‐smart and smart hydrogels as vitreous substitutes, where Lin et al. have compiled the findings in a comprehensive review.^[187]^

Santhanam et al. have engineered a smart in situ forming hydrogel based on gellan (*i.e.* an analog of collagen) and poly(methacrylamide‐co‐methacrylate (*i.e.* an analog of HA; poly(MAM‐co‐MAA).^[188]^ The optical, biochemical, and physical were similar to those of the native vitreous and was shown to remain intact, without degradation signs, for up to 4 weeks in vitro.^[188]^ Moreover, the smart hydrogel showed in vitro biocomptability with human RPE cells and fibroblasts, with sol‐gel transition temperatures ranging from 35.5 to 43°C.^[188]^ Extending their work on the two‐component smart hydrogel on an in vivo rabbit model, Laradji et al. have shown full biocompatibility, where rabbits treated with the thiolated gellan and poly(MAM‐co‐MAA‐co‐BMAC hydrogel showed no signs of retinal toxicity through similar optical coherence tomography imaging results as the control group treated with the SO vitreous substitute.^[189]^ Moreover, their thermoresponsive properties were shown to be optimal for clinical use: the smart hydrogel exhibits a viscous form at 45°C, with rapid gelation once body temperature is reached following injection.^[189]^ At the moment of gelation, the poly(MAM‐co‐MAA‐co‐BMAC hydrogel was able to form a vitreous similar to the native vitreous in terms of biochemical properties—owing it to its particular 3D rigid double helix compact structure.^[189]^ Additional copolymer smart hydrogels have shown great potential in preclinical studies as vitreous substitutes. Using copolymer hydrogels, Wang et al. demonstrated that the copolymerization of *N*‐acryloyl glycinamide (NAGA) and carboxybetaine acrylamide yields a hydrogel that is extrudable, given its sheer‐thinning behavior, and with self‐healability properties.^[190]^ In fact, at a physiological temperature, PNAGA‐PCBAA hydrogels are capable of reforming molecular bonds, thus repairing their structure. These are compelling results given their great potential as vitreous substitutes for vitreoretinal diseases. In another study, Xue et al. showed that the poly[(R)‐3‐hydroxybutyrate‐(R)‐3‐hydroxyhexanoate] (PHBHx)‐based polyurethane thermogel could also be an ideal alternative given the absence of retinal inflammation in rabbits for over 6 months, its native‐like refractive index, and its great physiochemical properties.^[191]^ Similarly, Zhang et al. have demonstrated the possibility for long‐term use of smart hydrogels: intravitreal injection of polytetrahydrofuran (PTHF)‐based thermosensitive hydrogel, generated through polyurethane reaction, was shown to still maintain its polymeric networks after 3 months^[192]^ Similar to these outcomes, numerous recent advances have outlined the importance, biocompatibility, and/or native vitreous‐like properties of smart hydrogels, such as the hydroxypropyl chitosan crosslinked with alginate dialdehyde hydrogel,^[193]^ the enzymatically crosslinked silk‐gelatin and HA‐based hydrogels,[^194^, ^195^, ^196^] the poly(propylene glycol) (PPG) and poly(ε‐caprolactone) (PCL) crosslinked polymer with urethane bonds (termed EPC hydrogel),^[197]^ PEG‐based smart hydrogels,^[198]^ poly(acrylamide co‐acrylic acid)‐based hydrogel,^[199]^ and supramolecular in situ gelling hydrogels.[^190^, ^197^, ^200^]

Despite these promising results, challenges and limitations remain for the use of smart hydrogels as vitreous substitutes. Although not common, hydrogels were shown to trigger the immune system, which leads to retinal and subretinal space inflammation.^[201]^ These adverse effects are mainly attributable to the small molecule crosslinkers—essential parts of hydrogels, where their absence enhances the degradation rate. Toxicity could be leveraged by prior crosslinking of the hydrogel and a prolonged waiting period before injection, therefore lowering the levels of free monomers and crosslinkers. The difficulty then resides in the ophthalmologist, who disposes of a shorter period frame to proceed with the intravitreal injection. Furthermore, proper sterilization sets back the use of hydrogels clinically; excessive heat could destroy the chemical bonds and physical structure of hydrogels.^[181]^ A balance between safety and efficacity has yet to be reached, and further studies should investigate the clinical applications of this innovative class of vitreous substitutes.

## 4 CONCLUSION

Recent advances in smart hydrogel technologies have certainly been a pivotal tool for the development of novel therapies and hydrogel‐based DDS. Most studies have shown their biocompatibility and power in treating ocular diseases in preclinical studies. However, an enormous step must be overcome in order to further apply these technologies in the clinical setting with ophthalmologists. Further clinical studies are needed in order to assess a more accurate pharmacokinetic, as well as long‐term drug safety.

## CONFLICT OF INTEREST STATEMENT

All authors have no conflicts of interest.

## Data Availability

Data sharing is not applicable to this article as no new data were created or analyzed in this study.
